# A reversible light- and genotype-dependent acquired thermotolerance response protects the potato plant from damage due to excessive temperature

**DOI:** 10.1007/s00425-018-2874-1

**Published:** 2018-03-08

**Authors:** Almudena Trapero-Mozos, Laurence J. M. Ducreux, Craita E. Bita, Wayne Morris, Cosima Wiese, Jenny A. Morris, Christy Paterson, Peter E. Hedley, Robert D. Hancock, Mark Taylor

**Affiliations:** 1 School of Biology, Biomolecular Sciences Building, University of St Andrews, North Haugh, St Andrews, Fife, Y16 9ST UK; 2 Cell and Molecular Sciences, The James Hutton Institute, Invergowrie, Dundee, DD2 5DA UK; 3 College of Arts and Sciences, Misericordia University, 301 Lake Street, Dallas, PA 18612 USA

**Keywords:** Acquired thermotolerance, Electrolyte leakage, Heat tolerance, Potato, Redox couples, Yield

## Abstract

A powerful acquired thermotolerance response in potato was demonstrated and characterised in detail, showing the time course required for tolerance, the reversibility of the process and requirement for light.

Potato is particularly vulnerable to increased temperature, considered to be the most important uncontrollable factor affecting growth and yield of this globally significant crop. Here, we describe an acquired thermotolerance response in potato, whereby treatment at a mildly elevated temperature primes the plant for more severe heat stress. We define the time course for acquiring thermotolerance and demonstrate that light is essential for the process. In all four commercial tetraploid cultivars that were tested, acquisition of thermotolerance by priming was required for tolerance at elevated temperature. Accessions from several wild-type species and diploid genotypes did not require priming for heat tolerance under the test conditions employed, suggesting that useful variation for this trait exists. Physiological, transcriptomic and metabolomic approaches were employed to elucidate potential mechanisms that underpin the acquisition of heat tolerance. This analysis indicated a role for cell wall modification, auxin and ethylene signalling, and chromatin remodelling in acclimatory priming resulting in reduced metabolic perturbation and delayed stress responses in acclimated plants following transfer to 40 °C.

**Electronic supplementary material:**

The online version of this article (10.1007/s00425-018-2874-1) contains supplementary material, which is available to authorized users.

## Introduction

Heat stress in crops causes profound negative impacts on global agriculture and food security in the absence of adequate adaptation. For example, it is estimated that over the last three decades heat stress has reduced global yields of wheat and maize by 5.5 and 3.8%, respectively, with even greater losses in specific regions (Lobell et al. 2011). In view of the severity of this problem, research has focussed on understanding the mechanisms underlying the response of plants to heat stress, with the long-term objective of translating findings to improved breeding strategies for crop plants.

Studies in the model plant Arabidopsis have identified four major thermotolerance types (Yeh et al. 2012). These responses include basal thermotolerance, short- and long-term acquired thermotolerance, and thermotolerance to moderately high temperatures. This ‘thermotolerance diversity’ means that multiple phenotypic assays are essential for fully describing the functions of genes involved in heat stress responses. In crop plants, a recent meta-analysis of temperature responses of developmental processes, involving the world’s most important staple crops including potato, suggested that despite centuries of breeding, high-temperature tolerance has not been significantly improved (Parent and Tardieu 2012). Targeted breeding approaches are hampered by a lack of fundamental knowledge regarding the molecular mechanisms by which plants perceive and translate high temperature into relevant acclimation responses.

Higher plants exposed to excess heat exhibit a characteristic set of cellular and metabolic responses required for survival (Guy 1999). The early effects of thermal trauma include structural alterations in chloroplast protein complexes, reduced enzyme activity, changes in the organisation of cellular structures and membrane functions, accompanied by a decrease in the synthesis of normal proteins and an accelerated transcription and translation of heat shock proteins (HSPs). The detrimental effects of heat on the photosynthetic apparatus are also associated with the production of potentially harmful reactive oxygen species (ROS). Although ROS can be a direct cause of cellular damage, they also play a key role as molecular signals, linking plant metabolism to environmental stresses. Subsequently, ROS production contributes to the transduction of the heat signal and expression of heat shock protein genes (Bita and Gerats 2013).

Acquired thermotolerance is a powerful adaptive response that enables plants to survive temperatures that are lethal when plants are not primed (Hilker et al. 2016). The acquired thermotolerance response has been described for a number of plant species (Song et al. 2012). Many temperature profiles have been reported to induce thermotolerance, with different plant species having particular requirements for acquiring heat tolerance.

Recent studies have started to address molecular mechanisms that underpin acquired thermotolerance. Global transcriptional profiles during different heat treatment regimes in Arabidopsis have been described (Larkindale and Vierling 2008). Direct exposure to high temperature elicited a very different transcriptional response compared with plants that were acclimated at a moderately high temperature prior to high-temperature treatment. Furthermore, different acclimation treatments resulted in different transcriptional profiles, although the plants appeared to have a similar degree of priming to the high-temperature condition.

A well-characterised aspect of acquired thermotolerance is the production of HSPs. During acclimation, plants induce substantial transcription and translation of HSPs that act as molecular chaperones to protect cellular proteins against irreversible heat-induced denaturation and to facilitate refolding of heat-damaged proteins. A well-defined response of plants to high temperatures involves the enhanced production of these molecular chaperones, including heat shock proteins HSP100 and HSP70, which are ATPases, and Class I and Class II small heat shock proteins (sHSPs). These molecular chaperones are believed to prevent or reverse the inactivation of heat-sensitive proteins, thereby restoring cellular protein homeostasis disrupted by environmentally challenging conditions (McLoughlin et al. 2016).

A key issue surrounding acclimation responses is how long the plant remains acclimated once priming has taken place and the plant returns to non-stress temperatures. This phenomenon is known as heat stress memory and, although an important element in the heat stress response, only recently are components of the mechanism emerging (Hilker et al. 2016).

Potato is the third most important food crop in the world after rice and wheat. More than a billion people worldwide eat potato, and global crop production exceeds 300 MT (Birch et al. 2012). Yet, this crop is particularly vulnerable to increased temperature, considered to be the most important uncontrollable factor affecting growth and yield. Elevated temperature is known to affect multiple processes in potato plant physiology. Tuber development is sensitive to elevated temperature as the tuberisation signal is inhibited at higher temperatures (Ewing 1981). Carbon transport to sink organs in the potato plant is very temperature sensitive, with less incorporation of assimilated carbon into starch in the tuber at elevated temperatures (Hancock et al. 2014). Photosynthetic performance is adversely affected alongside tuber formation, as heat leads to chlorophyll loss and reduced CO_2_ fixation (Reynolds et al. 1990). Here, we describe acquired thermotolerance responses in potato, whereby treatment at a mildly elevated temperature primes the plant for more severe heat stress. We define the time course for acquiring thermotolerance and demonstrate that light is essential for the process. In all four commercial tetraploid cultivars that were tested, acquisition of thermotolerance by priming was required for tolerance at elevated temperature. Accessions from several wild species and diploid genotypes did not require priming for heat tolerance under the test conditions employed, suggesting that useful variation for this trait exists. Physiological, transcriptomic and metabolomic approaches were employed to elucidate potential mechanisms that underpin the acquisition of heat tolerance and we describe differences in response to severe heat stress between acclimated and non-acclimated plants.

## Materials and methods

### Plant material and growth conditions

The commercial tetraploid potato cultivars Bintje, Atlantic, Desiree and Norchip were obtained from Science and Advice for Scottish Agriculture, Edinburgh, UK, and accessions representative of eight wild diploid *Solanum* species were obtained from the Commonwealth Potato Collection (Bradshaw and Ramsay 2005). Diploid genotypes from a well-characterised biparental diploid potato population, 06H1 (Prashar et al. 2014), were also used.

Plants were propagated from stem cuttings in 90 mm Petri dishes containing MS medium (Murashige and Skoog 1962) supplemented with 20 g l^−1^ sucrose and 8 g l^−1^ agar at 18 ± 4 °C, 16 h light, 8 h dark, and light intensity 100 μmol m^−2^ s^−1^. Four weeks after subculture, in vitro plantlets were transferred to 10 cm diameter pots containing a standard compost mix. Plants were grown in a glasshouse maintained at a daytime temperature of 18 °C and a nocturnal temperature of 15 °C. Light intensity (photosynthetic photon flux density) ranged from 400 to 1000 μmol m^−2^ s^−1^.

### Thermotolerance assay

Three weeks after transfer from tissue culture to the glasshouse, plants were moved to growth chambers and maintained at 18 °C (control treatment, referred to in text and figures as C) for 1 week at a relative humidity of 60% and a day length of 12 h with a light intensity of 150 μmol m^−2^ s^−1^. To induce thermotolerance, plants were then transferred to 25 °C under constant light or dark for different periods of time (30 min, 1, 2, 6, 12, 24 or 48 h). The degree of thermotolerance was then estimated by electrolyte leakage assays following exposure of plants to heat stress temperatures of 40 °C for 12, 24 or 48 h. To test how long the plants remain acclimated upon return to non-stress temperatures, an additional recovery assay was implemented to follow the heat stress (HS) treatment consisting of various episodes of recovery at 18 °C for 6, 12, 24, and 48 h and re-exposure to HS.

### Electrolyte leakage assay

Membrane damage was assessed using an electrolyte leakage assay (Campos et al. 2003). Four replicate samples of three 10 mm leaf discs from the fourth node were punched from a single leaf per sample assayed and placed in a 50 ml tube. The discs were washed twice with de-ionised water, blotted dry and returned to the tube with 5 ml of de-ionised water. Following gentle shaking for 1 h at 29 °C, 25 ml de-ionised water was added and the initial conductivity was measured using a conductivity meter (Model HI99300, Hanna Instruments Ltd, Leighton Buzzard, UK). Samples were autoclaved, and total conductivity was determined after cooling to room temperature. The percentage of injury (membrane damage) was calculated as: Initial conductivity × 100/total conductivity.

### Chlorophyll fluorescence measurements

Measurements of maximum quantum yield of PSII (Fv/Fm) were conducted using a Handy PEA fluorimeter (Hansatech Instruments Ltd., Norfolk, UK). Terminal leaflets on the fourth node were dark adapted for 20 min prior to the collection of fluorescence parameters following a saturating light pulse of 3000 μmol m^−2^ s^−1^.

### RNA and cDNA synthesis and qRT-PCR

Compound leaves of the fourth node were harvested from four replicate plants during the period of thermotolerance acquisition (0, 2, 6, 12 h) and following transfer of acclimated and non-acclimated plants to 40 °C for different periods of time (2, 6, 12, 24 h). RNA was extracted from potato leaves as described previously (Ducreux et al. 2008). First-strand cDNA templates were generated by reverse transcription, using TaKaRa RNA to cDNA EcoDry™ beads (http://www.clontech.com). Potato elongation factor1-alpha (EF1α) primers were used as a control. Quantitative PCR was performed as previously described (Trapero-Mozos et al. 2017).

### Microarray methods

A custom Agilent microarray was designed to the predicted transcripts from assembly v. 3.4 of the DM potato genome as described by Morris et al. (2014). The experimental design and complete datasets are available at ArrayExpress (http://www.ebi.ac.uk/arrayexpress/; accession numbers; E-MTAB-5857 and E-MTAB-5863). Briefly, a single-channel microarray design was utilised with leaf RNA samples all labelled with Cy3. RNA labelling and downstream microarray processing were performed as recommended using the Low Input Quick Amp Labelling kit (v 6.5; Agilent). Following microarray scanning using an Agilent G2505B scanner, data were extracted from images using Feature Extraction [FE (v. 10.7.3.1)] software and aligned with the appropriate array grid template file (033033_D_F_20110315). Intensity data and QC metrics were extracted using the recommended FE protocol (GE1_107_Sep09). Entire FE datasets for each array were loaded into GeneSpring (v. 7.3; Agilent) software for further analysis. Data were normalised using default single-channel settings: intensity values were set to a minimum of 0.01, data from each array were normalised to the 50th percentile of all measurements on the array, and the signal from each probe was subsequently normalised to the median of its value across all samples. Unreliable data flagged as absent in all replicate samples by the FE software were discarded. Statistical filtering of data for each experiment was performed independently using two-way analysis of variance (ANOVA; *P* ≤ 0.05) for the factors ‘time’ and ‘acclimation state’, with Benjamini and Hochberg multiple testing correction.

### Metabolite profiling by gas chromatography/mass spectroscopy (GC/MS)

GC/MS analysis was performed on extracts from three biological replicates per treatment essentially as described by Hancock et al. (2014). The compound leaf from the fourth node was snap frozen in liquid nitrogen and then lyophilised. 100 ± 5 mg of dried material was weighed into glass tubes and extracted in 3 ml methanol for 30 min at 30 °C with agitation (1500 rpm). 0.1 ml each of polar (ribitol 2 mg ml^−1^) and non-polar (nonadecanoic acid methylester 0.2 mg ml^−1^) internal standard and 0.75 ml distilled H_2_O were added and extraction continued for a further 30 min as described. Next, 6 ml chloroform was added and extraction continued for 30 min under increased agitation at 2500 rpm. Phase separation was achieved by the addition of a further 1.5 ml of water and centrifugation at 1000*g* for 10 min. Polar metabolites were oximated by reaction with methoxyamine hydrochloride (20 mg ml^−1^ in anhydrous pyridine) at 50 °C for 4 h and then converted to trimethylsilyl derivatives following incubation with *N*-methyl, *N*-trimethylsilyl trifluroacetamide at 37 °C for 30 min. Non-polar metabolites were subjected to methanolysis in 1% (v/v) methanolic H_2_SO_4_ at 50 °C for 16 h. Following neutralisation with aqueous KHCO_3_ and phase separation, the solvent fraction was dried and subjected to trimethylsilylation as described for the polar fraction. Metabolite profiles for the polar and non-polar fractions were acquired following separation of compounds on a DB5-MSTM column (15 m × 0.25 mm × 0.25 μm; J&W, Folsom, CA, USA) using a Thermo-Finnigan DSQII GC/MS system. Samples were injected and held at 100 °C for 2.1 min, a temperature gradient of 25 °C min^−1^ was applied to 320 °C, and the temperature was then held for 3.5 min prior to oven cooling and the next injection. Peak identification was achieved by coelution and matched mass spectra with known standards for named compounds and data were processed using Xcalibur software.

### Extraction and quantification of oxidised and reduced ascorbate and glutathione

Potato leaves were harvested, rapidly frozen in liquid nitrogen and stored at – 80 °C until analysis. Samples were subsequently extracted in 0.2 M HCl at a ratio of 100 mg material ml^−1^. Oxidised and reduced ascorbate and glutathione were quantified using the method described by Queval and Noctor (2007).

## Results

### Acquired thermotolerance in tetraploid potato cultivars

**Fig. 1:**
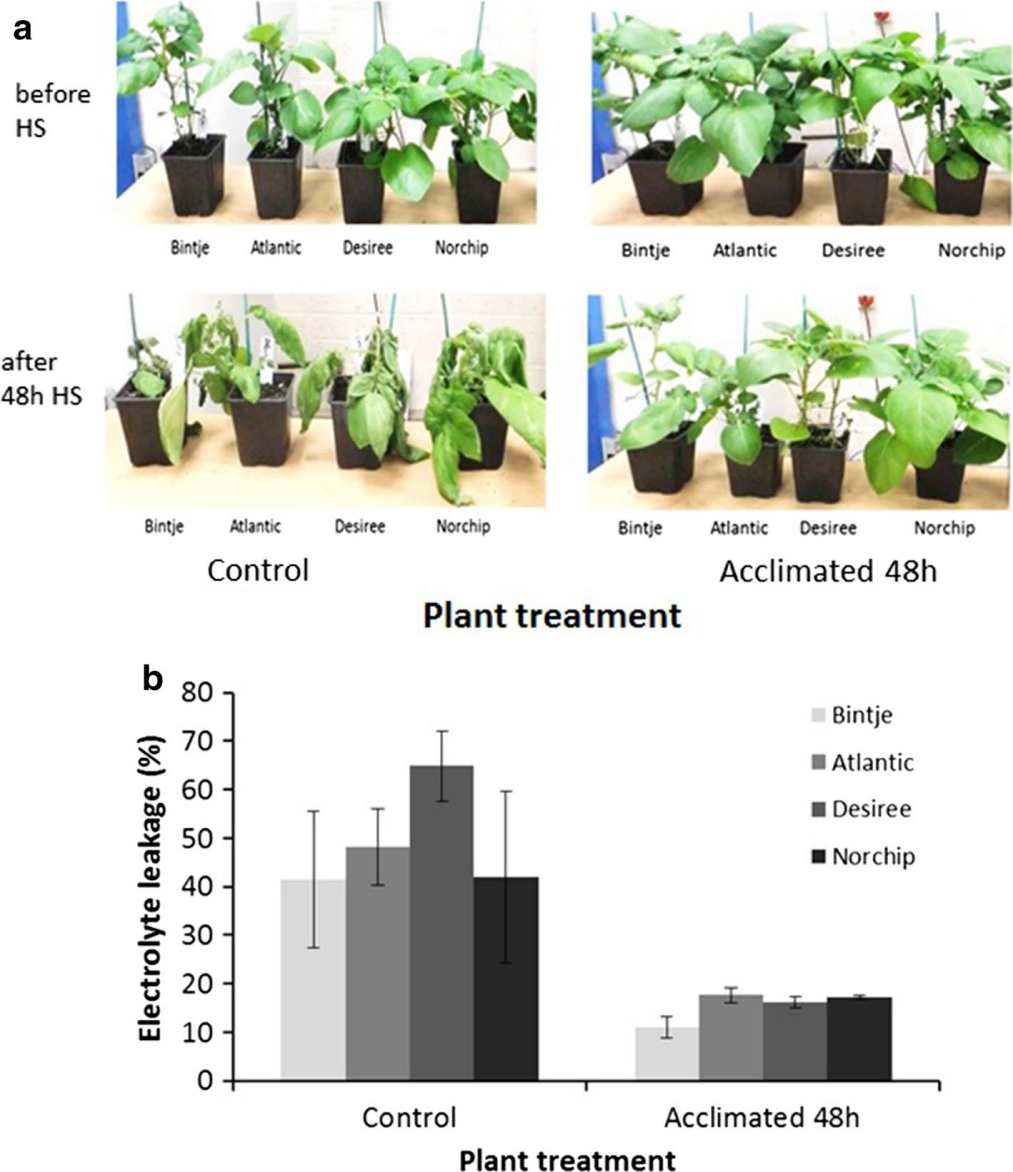
Acquisition of thermotolerance in commercial tetraploid cultivars. **a** Visual assessment of commercial potato cultivars kept at 18 °C (control) or acclimated for 48 h at 25 °C, before and after exposure to 40 °C heat stress (HS) for 48 h. **b** Relative electrolyte leakage in commercial potato cultivars kept at 18 °C or acclimated for 48 h at 25 °C after exposure to 40 °C heat stress for 48 h. Error bars represent the standard error of the mean (*n* = 3)

Four tetraploid commercial potato cultivars (Bintje, Atlantic, Desiree and Norchip) were selected to examine the effects of acquired thermotolerance on the response to elevated temperature. Plants were grown from tissue culture and maintained under glasshouse conditions for 3 weeks following subculture. Plants were then maintained in growth chambers at 18 °C for a period of 7 days under a day length of 12 h. Plants were then divided into two groups that were either maintained at 18 °C or subjected to a mildly elevated temperature of 25 °C. The light, temperature and watering conditions during these treatments are described in Online Resource S1. Following high-temperature exposure (40 °C), plants previously exposed to 18 °C immediately prior to transfer exhibited wilting and necrosis (Fig. 1a). On the contrary, those that were acclimated at 25 °C for 48 h were turgid and healthy after exposure to 40 °C (Fig. 1a). The visual phenotype was supported by measurements of electrolyte leakage (Fig. 1b). Damage in plants that had undergone treatment at 25 °C prior to transfer to 40 °C were significantly lower than in plants that had not been exposed to 25 °C as determined by ANOVA for acclimation treatment (*P *< 0.001). On the contrary, we were unable to discern differences between cultivars (*P *= 0.446) or any interaction between cultivar and acclimation treatment (*P *= 0.576).

### Detailed characterisation of acquired thermotolerance in the cultivar Desiree

**Fig. 2:**
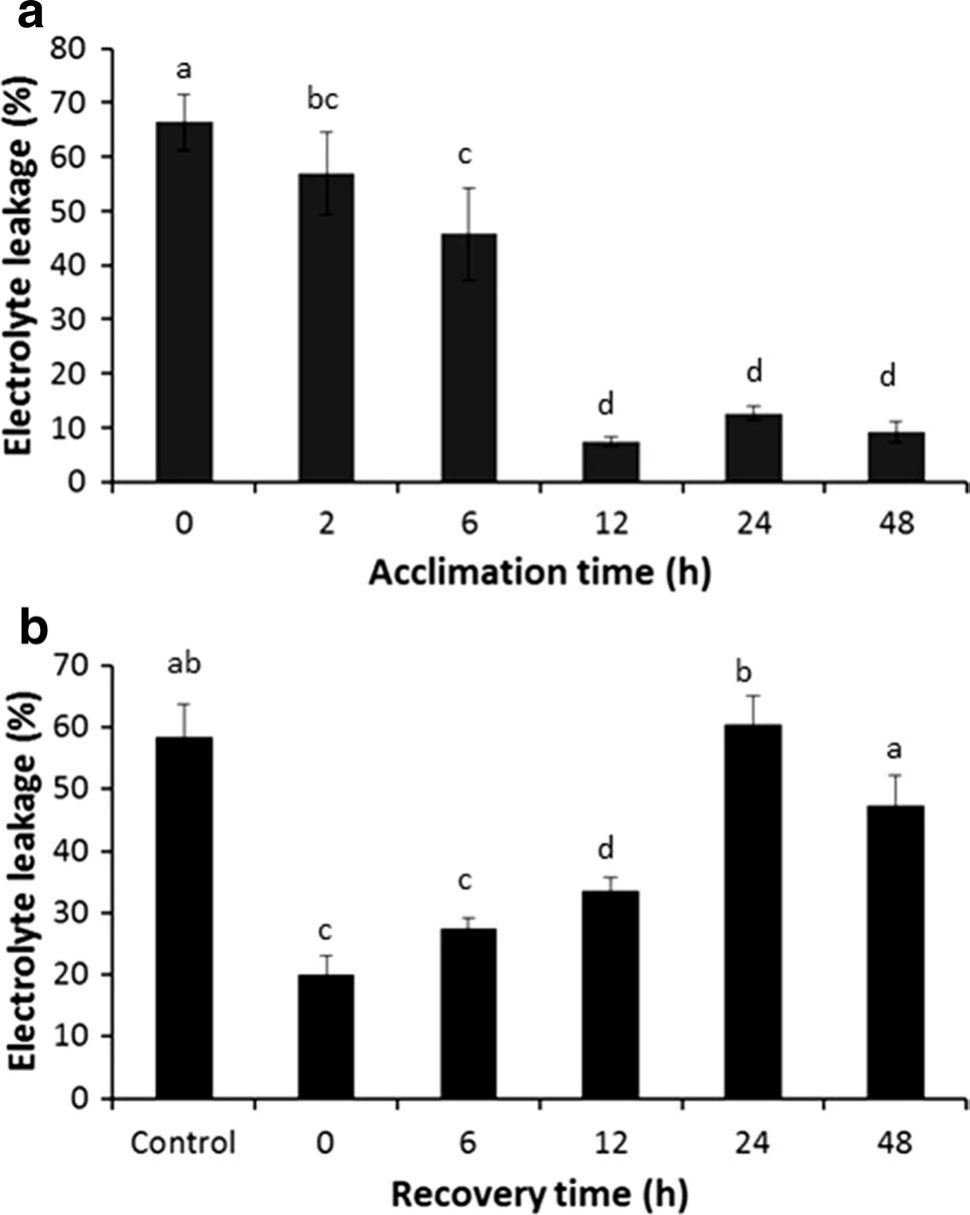
Thermotolerance acclimation response in the tetraploid cultivar Desiree. **a** Relative electrolyte leakage after exposure to 40 °C heat stress for 24 h following exposure to acclimation at 25 °C for different periods of time. Data are presented as mean ± SE, *n* = 8. Values that were significantly different as estimated by one-way ANOVA with Fisher’s protected LSD test are indicated by different letters. **b** Relative electrolyte leakage in plants exposed to 40 °C heat stress following acclimation at 25 °C for 12 h, followed by recovery at 18 °C for the time indicated prior to heat stress exposure. Control plants were only exposed to 18 °C. Data are presented as mean ± SE, *n* = 6. Values that were significantly different as estimated by one-way ANOVA with Fisher’s protected LSD test are indicated by different letters

After 48 h treatment at 25 °C, all four cultivars tested had acquired thermotolerance. To further define the acquisition kinetics, time course experiments were conducted in the cultivar Desiree using electrolyte leakage assays to measure thermal damage to cell membranes. These assays demonstrated that thermotolerance was initiated after 2 h of exposure to 25 °C and that it was completed by 12 h (Fig. 2a).

Further experiments were designed to investigate how long plants maintained thermotolerance once it was acquired. Plants were treated for 12 h at 25 °C under light and then returned to 18 °C under constant light. After different time periods at 18 °C, plants were exposed to 40 °C and membrane damage assessed after 24 h (Fig. 2b). Based on the electrolyte leakage assay results, thermotolerance was maintained for at least 12 h after transfer back from 25 to 18 °C. For plants maintained at 18 °C for 24 h or longer, thermotolerance was lost, and membrane damage levels were similar to or higher than non-acclimated controls, indicating that acquired thermotolerance persisted for between 12 and 24 h.

**Fig. 3:**
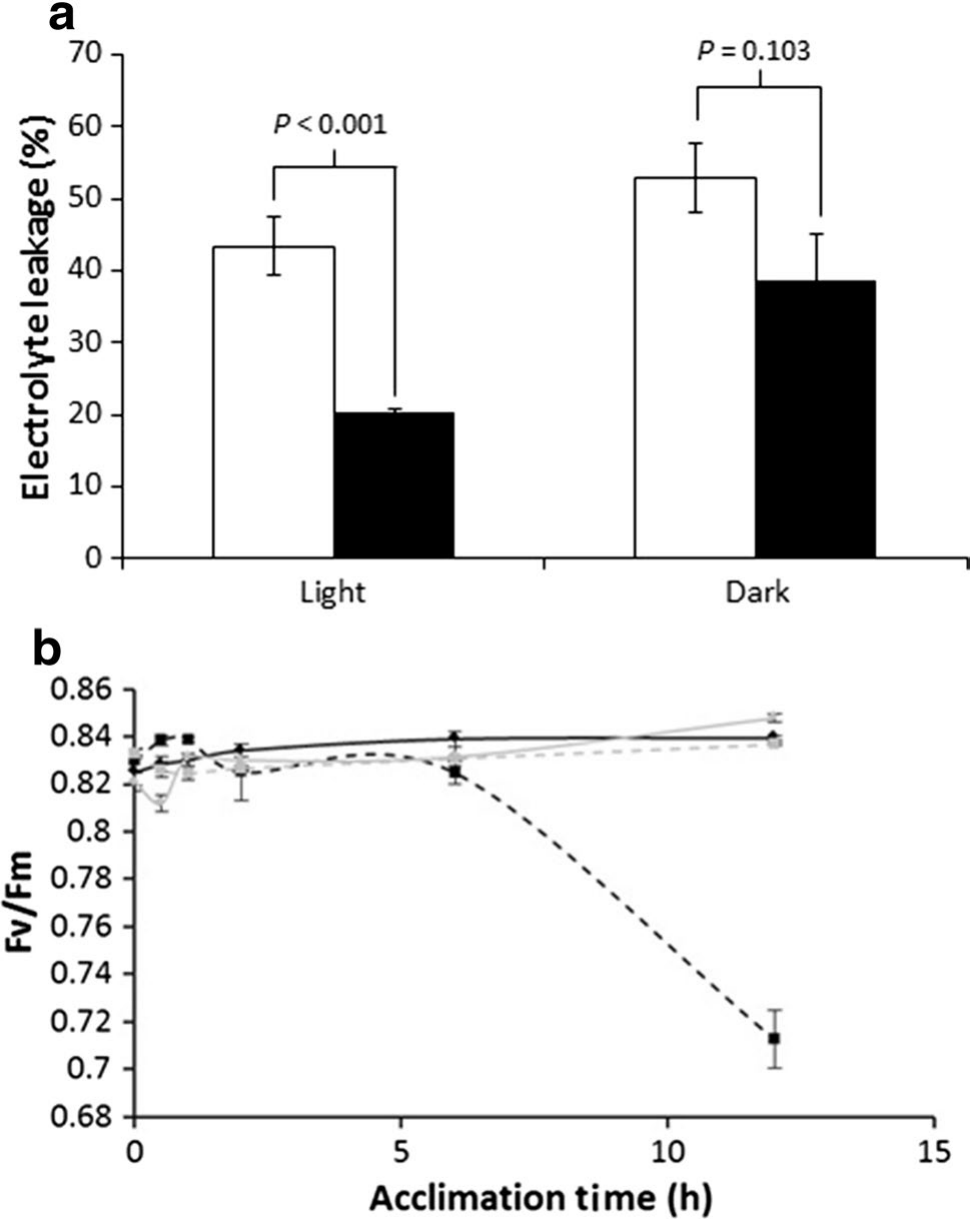
Influence of light on thermotolerance acclimation and maximum quantum yield of PSII in cultivar Desiree. **a** Relative electrolyte leakage after exposure to 40 °C heat stress for 24 h in plants kept at 18 °C (open square) or acclimated for 12 h at 25 °C (filled square) under constant light or dark. *P* values as determined by the Student’s *t* test are indicated and error bars represent the standard error of the mean (*n* = 6). **b** Maximum quantum yield of PSII during acclimation at 18 °C (solid lines) or 25 °C (dashed lines). Plants were either maintained in the light (light grey lines) or dark (black lines) and data are represented as mean ± SE, *n* = 6–8

We then investigated whether light was required to acquire thermotolerance. Plants were treated at 25 °C for 12 h either in complete darkness or under constant light, prior to transfer to 40 °C for 24 h with the first 12 h in the light and the second 12 h in the dark. Leaf membrane damage was assessed by electrolyte leakage. While plants incubated for 12 h at 25 °C in the light were clearly more resistant to high-temperature damage than those exposed to 18 °C in the light (Fig. 3a), plants that were exposed to 25 °C in the dark were no more resistant to high-temperature damage than those that had only experienced 18 °C (Fig. 3a). These data clearly indicate a necessity for light exposure in the acquisition of thermotolerance.

The requirement for light suggests that a chloroplast signal may be required for acquisition of thermotolerance. To further investigate the influence of moderately elevated temperature on photosynthetic performance, we quantified the maximum quantum yield of photosystem II (Fv/Fm) during acclimation at 18 or 25 °C in the dark or light. For the first 6 h of the experiment, all plants exhibited similar quantum yields of PSII fluctuating around 0.83 irrespective of the temperature or the presence or absence of light (Fig. 3b). These values are similar to those published for healthy plants without damage to PSII (Rykaczewska and Mańkowski 2015). Similarly, 12 h into the acclimation period plants kept at 18 °C in either the dark or the light continued to exhibit PSII quantum yields greater than 0.83 as did plants maintained in the light at 25 °C. On the contrary, plants maintained at 25 °C in the dark exhibited a significant reduction in PSII quantum yield indicative of dismantling or damage to the photosystem following 12 h exposure to this treatment (Fig. 3b).

Chloroplasts represent a significant source of ROS in the light, even when operating under optimal conditions (Foyer and Noctor 2003), suggesting that acclimation may require a chloroplast-derived oxidative signal. To assess whether the plants experienced oxidative stress during acclimation and high-temperature exposure, the major redox couples ascorbate (AsA)-dehydroascorbate (DHA) and reduced glutathione (GSH)-glutathione disulphide (GSSG) were quantified both during the acclimation phase at 25 °C and following exposure to temperature stress at 40 °C.

**Fig. 4:**
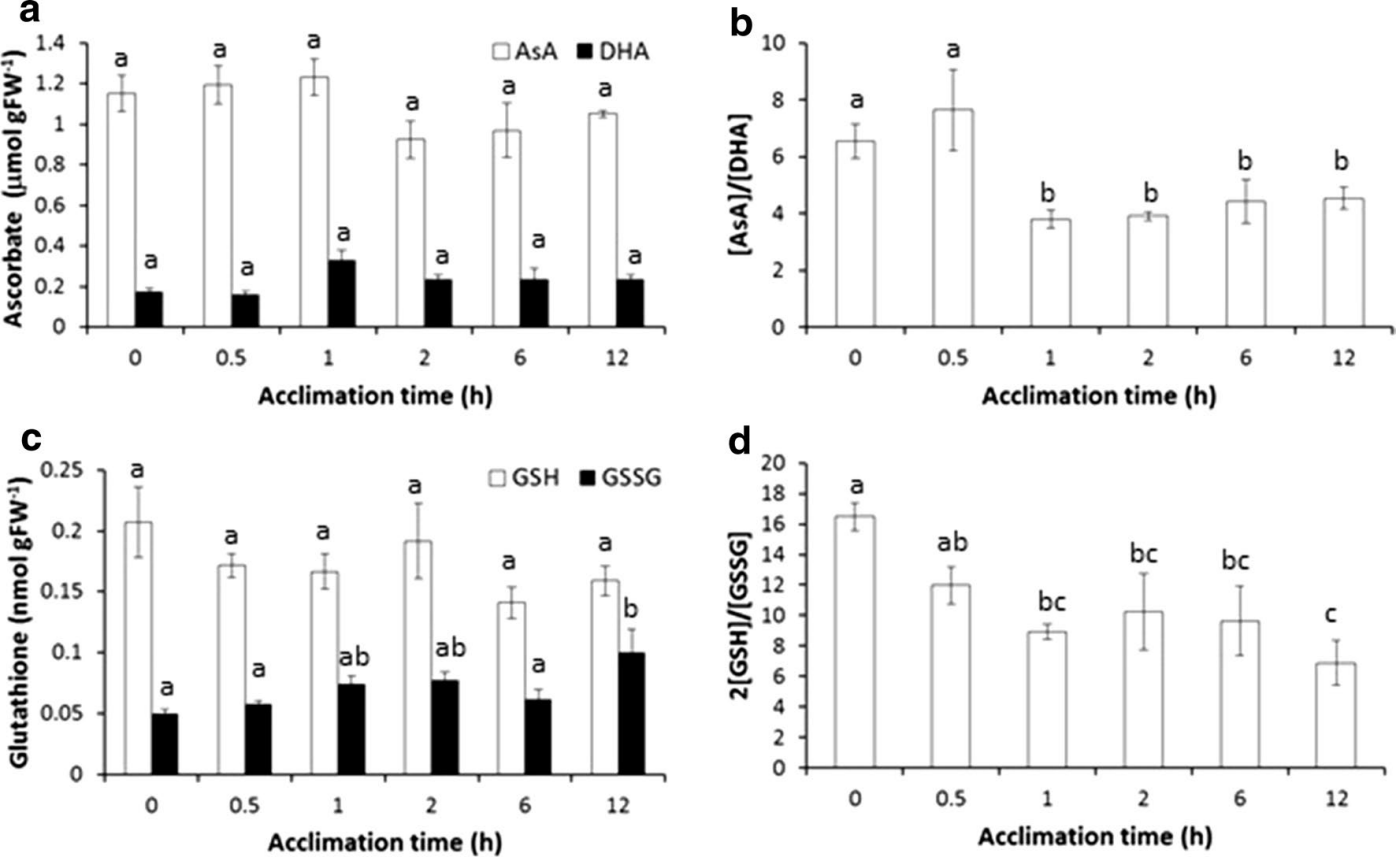
Response of antioxidant pools to acclimation at 25 °C in cultivar Desiree. Plants were transferred from 18 to 25 °C under constant light and leaves harvested at the times indicated. Ascorbate and glutathione were extracted and quantified as described. **a** Leaf content of reduced (AsA) and oxidised (DHA) ascorbate. **b** Ratio of AsA to DHA. **c** Leaf content of reduced (GSH) and oxidised (GSSG) glutathione. **d** Ratio of GSH to GSSG. All values are represented as mean ± SE (*n *= 3). Values labelled with the same letter were not significantly different (*P *< 0.05) as estimated by one-way ANOVA with Fisher’s LSD test

Acclimation at 25 °C was associated with increases in the oxidation state of both ascorbate and glutathione that was apparent following 1 h after transfer from 18 °C (Fig. 4). In the case of ascorbate, this was mainly due to a reduction in the pool of reduced ascorbate; however, this reduction was not statistically significant (Fig. 4a). Similarly, there was a trend towards lower content of reduced glutathione that was accompanied by a significant increase in the amount of oxidised glutathione 12 h after transfer to the acclimation temperature (Fig. 4c).

Leaf antioxidant content was also quantified 12 h after transfer to 40 °C (Table 1). Acclimated plants exhibited a significantly smaller total ascorbate pool than non-acclimated plants, which was reflected by a trend towards lower AsA and significantly lower DHA (Table 1). However, acclimated plants had a significantly more reduced ascorbate pool than non-acclimated plants. Acclimated plants additionally exhibited a trend towards a smaller and more reduced glutathione pool; however, results were not significant (Table 1).

**Table 1:** Redox buffers in Desiree leaves 12 h after transfer to 40 °C heat stress

	Non-acclimated	Acclimated
Total ascorbate	**1.33 ± 0.06**	**0.78 ± 0.07**
AsA (μmol gFW^−1^)	0.92 ± 0.09	0.68 ± 0.07
DHA (μmol gFW^−1^)	**0.41 ± 0.04**	**0.14 ± 0.01**
AsA/DHA	**2.31 ± 0.46**	**4.63 ± 0.19**
Total glutathione (nmol gFW^−1^)	0.29 ± 0.04	0.22 ± 0.01
GSH (nmol gFW^−1^)	0.22 ± 0.02	0.17 ± 0.00
2GSSG (nmol gFW^−1^)	0.07 ± 0.01	0.05 ± 0.00
GSH/2GSSG	3.26 ± 0.28	3.79 ± 0.29

Values are presented as mean ± SE (*n *= 3). Figures in bold represent significant differences between non-acclimated and acclimated leaves (*P *< 0.05) as determined using Student’s *t* test

### Variation in acquired thermotolerance responses in potato diploid genotypes including wild species

**Fig. 5:**
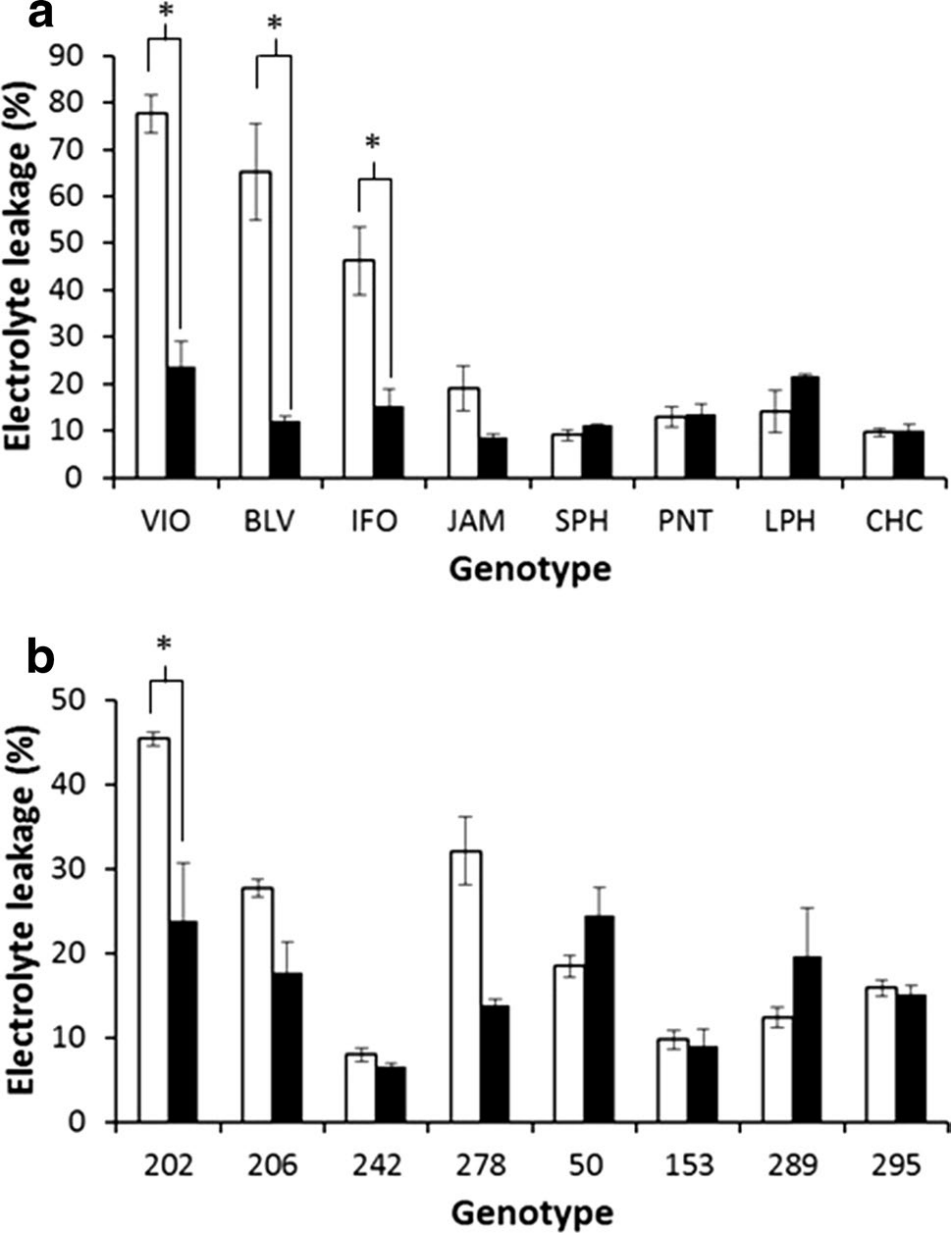
Variation in acquired thermotolerance in diploid potato wild species and the biparental diploid population 06H1. **a** Relative electrolyte leakage in control (open square) and acclimated (filled square) plants of *S. violaceimarmoratum* (VIO), *S. boliviense* (BLV), *S. infundibuliforme* (IFO), *S. jamesii* (JAM), *S. stenophyllidium* (SPH), *S. pinnatisectum* (PNT), *S. leptophyes* (LPH) and *S. chacoense* (CHC) following exposure to heat stress at 40 °C for 48 h (*n* = 3). **b** Relative electrolyte leakage in control (open square) and acclimated (filled square) heat-sensitive (202, 206, 242, 278) and heat-tolerant (50, 153, 289, 295) lines of the 06H1 population, following exposure to 40 °C heat stress for 48 h. Asterisks indicate significant differences between treatments as estimated by Student’s *t* test (*P *< 0.05)

Although acquired thermotolerance was demonstrated to be a powerful mechanism in the four commercial potato cultivars that were tested, we also investigated the degree of variation for this response in diverse potato genotypes. Accessions representative of eight wild potato species, maintained in the Commonwealth Potato Collection (Bradshaw and Ramsay 2005), were selected for this purpose. Plants were propagated in tissue culture and grown for 3 weeks at 18 °C, then either exposed to 25 °C or maintained at 18 °C for 12 h in the light. Plants were then immediately transferred to 40 °C and maintained for a further 48 h under light conditions as shown in Online Resource S1. Leaf membrane damage was then estimated by electrolyte leakage. For accessions from *S. violaceimarmoratum* (VIO)*, S. boliviense* (BLV) and *S. infundibuliforme* (IFO), there was a clear difference in membrane damage between plants that were treated at 25 °C and those transferred directly to 40 °C. As with the *S. tuberosum* cultivars, treatment at 25 °C resulted in acquired thermotolerance. Interestingly, for the accessions from *S. jamesii* (JAM)*, S. stenophyllidium* (SPH)*, S. pinnatisectum* (PNT)*, S. leptophyes* (LPH) and *S. chacoense* (CHC), there was no significant difference in leaf membrane damage at 40 °C whether the plants had been exposed to 25 °C or transferred directly from 18 °C (Fig. 5a). In the genotypes that did not require acclimation at 25 °C, membrane damage was relatively low (less than 20%) indicating a high degree of basal thermotolerance.

**Fig. 6:**
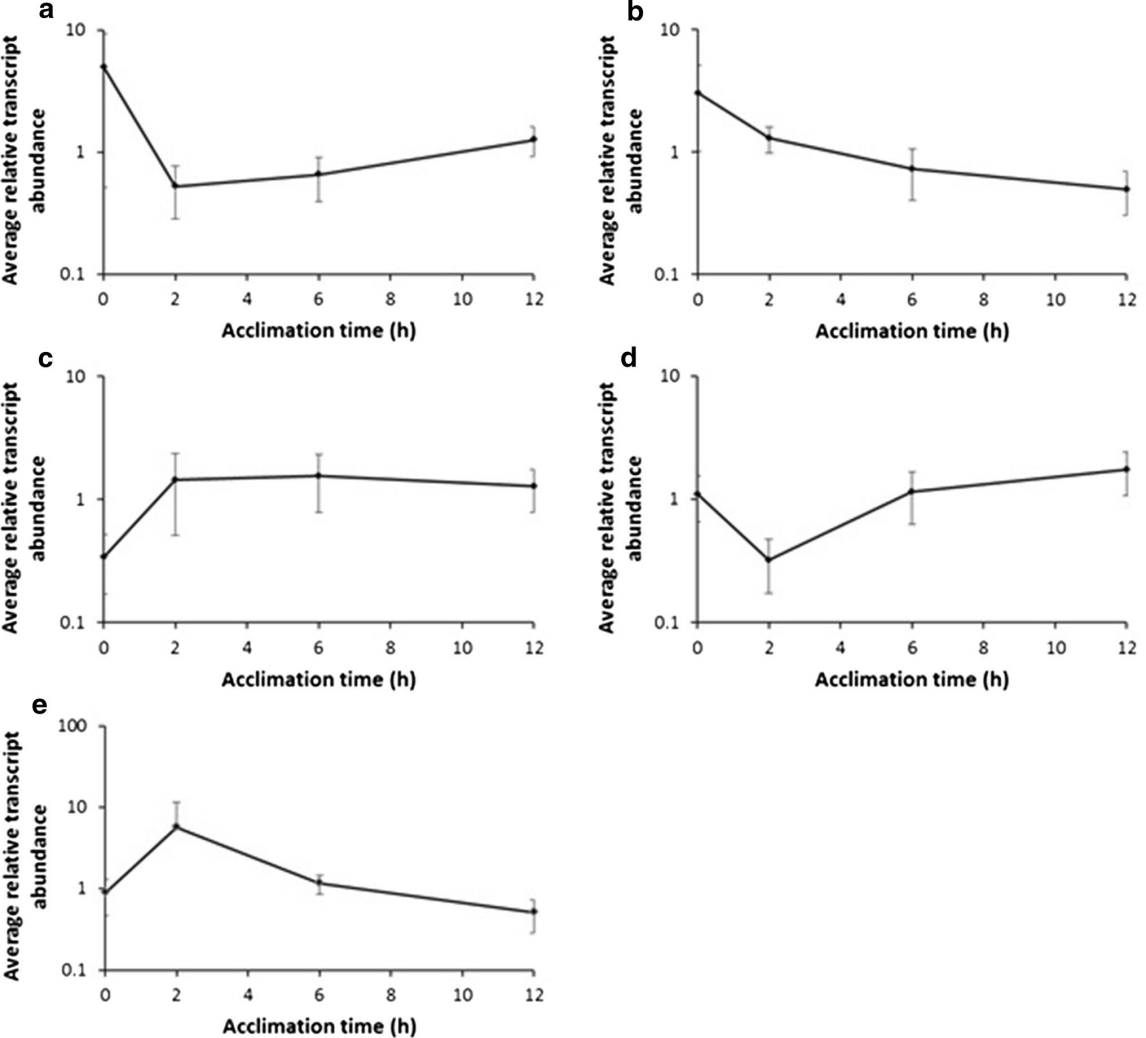
K-means clusters of gene expression during heat acclimation at 25 °C in the light. Transcripts that exhibited significant differences in abundance dependent on time at 25 °C were arbitrarily clustered into five patterns of expression using the k-means algorithm in GeneSpring. Lines in panels **a**–**e** show the average abundance of transcripts at each of the times sampled within clusters **a**–**e**, respectively, and error bars indicate standard deviation

The acquired thermotolerance response was also investigated in genotypes from a well-characterised biparental diploid potato population—06H1 (Prashar et al. 2014). Tuberisation in 180 genotypes from this population was assessed by measuring the tuber yield in a model nodal cutting tuberisation assay. The tuber yield was measured in cuttings maintained at 22 and 28 °C. Four genotypes that exhibited a major yield reduction at 28 °C (heat sensitive) and four genotypes where yield was maintained at the higher temperature (heat tolerant) were selected for characterisation of acquired thermotolerance (Online Resource S2). The acquired thermotolerance assessment was conducted as described for the wild species. For three out of four heat-sensitive genotypes, membrane damage at 40 °C was relatively high (> 20%) following transfer from 18 to 40 °C which contrasted with the heat-tolerant genotypes that exhibited low membrane damage (< 20%) upon direct transfer (Fig. 6b). However, genotype 242 identified as heat sensitive in the yield assay (Online Resource S1) also exhibited very low membrane damage irrespective of prior acclimation at 25 °C (Fig. 5b).

### Gene expression and metabolite changes during acclimation

Transcriptomic analysis was performed on potato leaf samples using an Agilent 60-mer microarray (“Materials and methods”) containing probes representing all 39031 protein coding genes predicted from the potato genome sequence (Potato Genome Sequencing Consortium 2011).

Changes in transcript level were measured during the acclimation time course following transfer from 18 to 25 °C over 12 h during the period of acquisition of thermotolerance (Fig. 2a). Analysis of variance, using a *P* value threshold of 0.05, identified 492 genes that were significantly different in abundance dependent on the time plants were subjected to treatment at 25 °C. K-means clustering of this set of genes was used to identify five main patterns of gene expression during the 12 h acclimation period (Fig. 6). To highlight specific biological functions of transcripts within each of the K-means clusters, they were classified using the MapMan tool (Thimm et al. 2004; Online Resource S3). Cluster 1 is characterised by a sharp decrease in transcript level over 2 h followed by a steady recovery, although transcript abundances remained lower at the end of the acclimation period than at the beginning. Prominent in this group are genes that respond to auxins (bin 17.2.3) and ethylene (bin 17.5.2). There are also a high proportion of genes involved in regulation of transcription (bin 27.3), particularly those encoding AP2/ethylene response element binding proteins (EREBP), zinc finger family proteins, and MYB and WRKY transcription factors. Cluster 2 transcripts exhibit a decrease in abundance throughout the acclimation period. Genes involved in photosynthetic light reactions, particularly those involved in chlorophyll A/B binding (bin 1.1.1.1), are represented in this group. Also of interest in this cluster are three genes encoding glutaredoxins (bin 21.4) and several genes in bin 28.1 that have a function in DNA synthesis/chromatin structure. This cluster also contains several transcripts associated with cell wall remodelling such as transcripts encoding a number of expansins and one encoding a pectin methylesterase. Cluster 3 transcripts exhibit a rapid increase in abundance over 2 h and then remain at a relatively constant level for the remainder of the acclimation time course. This relatively small cluster comprising only 37 transcripts was characterised by 12 transcripts encoding HSPs (bin 20.2.1). Further evidence for cell wall modification during heat acclimation was evident from the observation that this cluster also contains a number of transcripts encoding xyloglucan endotransglycosidases (bin 10.7) as well as one transcript encoding a β-xylosidase. Cluster 4 transcripts exhibited a significant decrease in abundance 2 h following transfer to 25 °C, but by 6 h transcript levels had fully recovered and were then maintained or slightly elevated at the end of the acclimation period (12 h). Fifteen of the 150 transcripts in this cluster were associated with cell wall synthesis or modification (bin 10). Seventeen transcripts encoded proteins involved in hormone metabolism or response, particularly auxin-responsive genes (bin 17.2.3), but also including transcripts related to cytokinin, ethylene, gibberellin and jasmonate metabolism and response. Cluster 5 transcripts exhibited a rapid increase in abundance 2 h after transfer to acclimatory temperature, followed by a steady decline towards the zero time level over the next 10 h. This relatively small cluster of 63 transcripts included several transcripts associated with ethylene synthesis (bin 17.5.1), two transcripts related to ubiquitin-mediated protein degradation (bin 29.5.11.4.2) and transcripts related to calcium signalling (30.3). Seven transcripts encoded transcription factors of various groups, including AP2/EREBP, zinc finger and WRKY.

While the analysis outlined above provided an indication of the processes associated with thermal acclimation, we wished to identify the transcriptional changes that acted as markers of completion of the acclimation process. We hypothesised that the transcriptional changes that were unique at the 12 h time point would correlate with the functions that were primed to allow survival at 40 °C. To identify transcripts that were uniquely altered in abundance at 12 h, we compared gene expression differences between time points using volcano plots (Student’s *t* test/fold change). A Venn diagram of the resulting significant gene lists was constructed to identify gene expression changes specific to each time point (Online Resource S4). Using this approach, 77 genes were identified that were uniquely differentially expressed between the time zero and 12 h time points (Online Resource S5). Gene ontology analysis of this set of genes enabled the major functional categories to be identified (Online Resource S5). This list of transcripts shared similarities with those obtained using ANOVA. In the acclimated (12 h), compared to the non-acclimated (0 h) state, we observed a reduction in the abundance of transcripts encoding chlorophyll binding proteins (bin 1.1.1.1), cell wall modification (bin 10.7), glutaredoxins (bin 21.4) and DNA modification/chromatin structure (bin 28.1). Other ontologies in this list include genes related to protein degradation, auxin and ethylene signalling; however for these classes, some transcripts increase whereas others decrease.

To investigate metabolite changes, parallel samples were extracted in a two-phase solvent system and following derivatisation as tri-methyl silyl and methyl ester derivatives, respectively, the metabolite profiles of the polar and non-polar fractions were obtained by GC/MS (Online Resource S6). One-way analysis of variance revealed only seven polar compounds that exhibited significant changes in leaf abundance during acclimation (*P *< 0.05). We therefore relaxed significance criteria to *P *< 0.1, which resulted in the identification of 13 polar and 3 non-polar compounds. Altogether, seven of these compounds were not identified, leaving only nine identified compounds (7 polar, 2 non-polar) that exhibited significant changes during leaf acclimation (Online Resource S7). Two hours following transfer to the acclimatory temperature, the major nitrogen-containing amino acids asparagine and glutamine exhibited a transient rise in abundance that may be indicative of early protein turnover and realignment of *N* compartmentation. Allantoin, a breakdown product of purine nucleotides, was also significantly elevated 2 h after transfer to 25 °C. Mannitol and sorbitol were also elevated at the 2 h time point, perhaps to protect cell membranes and other macromolecules from temperature stress (Krasensky and Jonak 2012). At the 12 h time point, sucrose and galactinol were significantly elevated. In the non-polar fraction, the long chain fatty alcohol tetracosanol (C24) was significantly lower following 12 h acclimation than at other times, and nonacosanol (C29) was most abundant at 6 h.

### Gene expression and metabolite changes during exposure to high temperature

To understand the mechanisms by which acclimation resulted in protection of leaves at extreme temperature, gene expression patterns in leaves from acclimated and non-acclimated plants subsequently transferred to 40 °C were compared during a 24 h time course. Two-way analysis of variance on the basis of time and acclimation identified 9330 and 39 differentially expressed transcripts, respectively. A further 128 transcripts exhibited changes in abundance that were dependent on an interaction between time and temperature. Similarly, the largest number of metabolites was significantly altered in abundance by time at 40 °C (39), while abundances of 18 metabolites were significantly altered as a result of acclimation treatment and 11 showed variation dependent on an interaction between time and acclimation treatment (Online Resource S8). Analysis of metabolites significantly influenced by acclimation treatment or an interaction between acclimation treatment and time at 40 °C suggested greater metabolic perturbation in non-acclimated plants following transfer to 40 °C. This was indicated by the significantly higher amounts of sugars and organic acids, particularly at the earlier times following transfer (Online Resource S9). Threonate, a breakdown product of ascorbate, was also higher in non-acclimated plants shortly after transfer to 40 °C as were the stress-associated polyamines spermidine and putrescine. A number of non-polar membrane-associated compounds also exhibited differences between acclimated and non-acclimated plants; however, these differences were generally lower than those observed in the polar metabolites (Online Resource S9).

As an initial analysis of the processes affected by heat stress, we analysed the 9330 probes differentially expressed with respect to time via a Wilcoxon rank sum test to determine if specific ontologies were up- or downregulated relative to the median fold change of all genes (Usadel et al. 2006, Online Resource S10). According to this analysis, both acclimated and non-acclimated plants exhibited broadly similar transcriptional responses to high-temperature stress, with plants rapidly responding by upregulating the expression of transcripts associated with both heat and drought/salt stress within 2 h of exposure. Transcripts encoding heat shock factors were also rapidly induced within 2 h irrespective of acclimation treatment. Similarly, after prolonged exposure to 40 °C, the abundance of transcripts associated with light harvesting and electron transport, as well as downstream enzymes of the Calvin cycle, was significantly reduced as were transcripts associated with starch synthesis. Another common late response was the increase in abundance of transcripts encoding proteins from several transcription factor families, as well as transcripts associated with chromatin structure. Additionally, a number of transcripts exhibited divergent behaviour dependent upon acclimation treatment. For example, non-acclimated plants exhibited an initial increase in the abundance of transcripts associated with PSII and PSI that was not observed in acclimated plants. On the contrary, acclimated plants exhibited a significant increase in the abundance of transcripts encoding ribosomal proteins following 12 h exposure to temperature stress that was not observed in non-acclimated plants. Interestingly, the abundance of transcripts associated with ubiquitination and protein degradation was higher in acclimated compared with non-acclimated plants after 24 h exposure. However, both acclimated and non-acclimated plants exhibited a significant increase in the content of several amino acids following extended exposure to 40 °C (Online Resource S9), consistent with protein degradation and turnover.

**Fig. 7:**
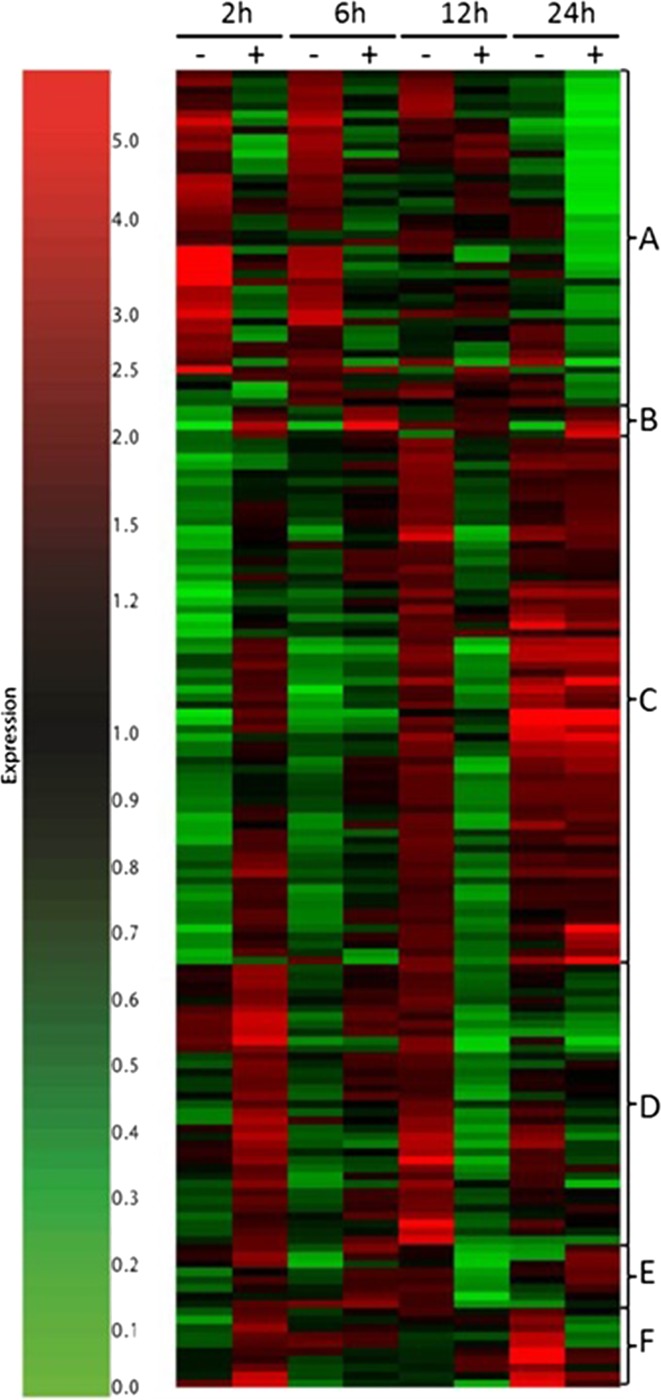
Heat map of relative transcript abundance in acclimated and non-acclimated potato leaves following different times of exposure to 40 °C. Leaves from acclimated (+) and non-acclimated (−) plants subsequently transferred to 40 °C were compared during a 24 h time course sampled at the times indicated. Transcripts that exhibited significant changes in abundance dependent on prior acclimation treatment or a treatment by time of exposure to 40 °C interaction (*n *= 128) was subjected to hierarchical tree clustering. The results are expressed as fold change of transcript abundance relative to transcript abundance immediately prior to transfer to 40 °C. Clusters described in the text are indicated as different letters to the right of the heat map

To further analyse specific differences in the transcriptional response of acclimated versus non-acclimated plants to exposure to temperature stress, we combined the expression data from the 39 probes that showed significant differential expression based on acclimation treatment with the data from the 128 probes that exhibited a time by acclimation interaction effect on expression. Transcript expression profiles were then subjected to hierarchical cluster analysis which indicated six major clusters of expression patterns (Fig. 7). Cluster A contained 42 probes that were highly expressed at the earlier time points (2 h, 6 h) in non-acclimated plants only. An analysis of gene ontology in this group using the MapMan tool (Thimm et al. 2004) revealed significant enrichment in categories associated with chromatin organisation, as emphasised by the observation that a large number of transcripts encoded histones (bin 28.1.3). Transcripts associated with biotic (bin 20.1) and abiotic (bin 20.2) stresses were also represented within this cluster (Online Resource S11). Cluster B contained only four transcripts that were highly expressed only in acclimated cells at all of the time points sampled. All of the transcripts within this cluster were assigned as having unknown function (bin 35). Cluster C was the largest of the clusters containing a total of 66 transcripts, 35 of which were assigned an unknown function. Transcripts in this cluster were highly expressed following 12 h exposure in non-acclimated plants and highly expressed at 24 h regardless of prior acclimation treatment. This cluster contained a number of transcripts encoding transcription factors (bin 27.3) including a transcript (DMT400084751) with a high degree of homology to *Arabidopsis* ZAT10 (at1g27730), a zinc finger protein involved in responses to photo-oxidative stress that is expressed in response to H_2_O_2_ accumulation (Rossel et al. 2007). Transcripts associated with protein degradation (bin 29.5) were additionally well represented within this group. Cluster D comprised 35 transcripts that were highly expressed in acclimated plants after 2 h exposure to high temperature, but that only increased in abundance in non-acclimated plants after 12 h exposure. The majority of transcripts encoded proteins of unknown function; however, two genes encoding HSPs were present within this cluster (bin 20.2.1). Cluster E comprised only eight transcripts that were expressed early in acclimated plants, but later in non-acclimated plants. Six of these transcripts were of unknown function, while the remaining two encoded a zinc finger protein and an MAP kinase, respectively. Cluster F comprised ten transcripts that were transiently expressed at 2 h in acclimated plants and then highly expressed in non-acclimated plants at 24 h. Five of the transcripts were of unknown function and two encoded transcripts with homology to the Arabidopsis cytokinin oxidase 5 (at1g75450) that catalyses the degradation of cytokinins, while a further transcript encoded an ethylene response factor.

### Heat shock protein expression during acclimation, stress and recovery from stress

To validate the gene expression changes identified in the acclimation array data, qRT-PCR assays were performed for three test genes. Very similar patterns of gene expression were observed using both techniques (Online Resource S12). The selected HSPs, used as test genes, were those previously implicated in acclimation processes (HSP17.6 and HSP101; McLoughlin et al. 2016) and a potato *HSC70* gene recently demonstrated to protect against elevated temperature in potato (Trapero-Mozos et al. 2017).

**Fig. 8:**
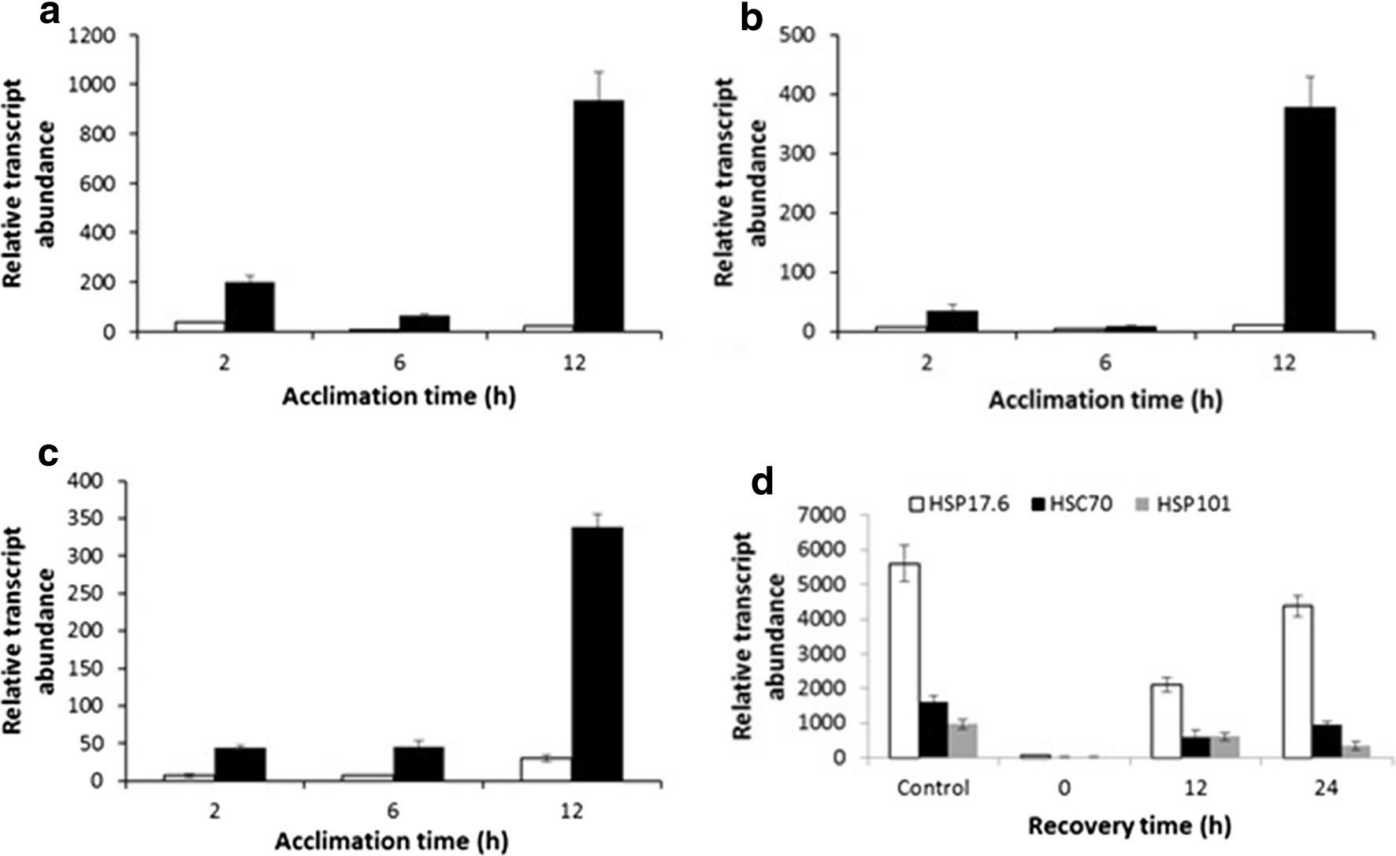
Influence of heat acclimation and heat stress on the expression of transcripts encoding heat shock proteins in potato leaves. The abundance of transcripts encoding HSP17.6 (**a**), HSC70 (**b**) and HSP101 (**c**) are indicated during acclimation at 25 °C in the light (open square) or dark (filled square), relative to transcript abundance immediately prior to transfer to acclimation temperature. **d** Abundance of transcripts encoding heat shock proteins in leaves exposed to 40 °C for 24 h. Data from unacclimated (control) or leaves that had been acclimated at 25 °C prior to recovery at 18 °C for various times are indicated. In all panels, data are represented as mean ± SE (*n* = 3)

In view of the potential importance of HSP gene expression during acclimation, the expression levels of these genes were compared during light and dark treatment at 25 °C. In leaves under continuous light at 25 °C, all three transcripts increased in abundance compared with samples from plants maintained at 18 °C, with HSP17.6-specific transcripts showing a large rapid increase in transcript level after 2 h (ca. 30-fold, Fig. 8a). HSC70 and HSP101 transcript levels increased more gradually over the 12 h time course to give levels 10- to 32-fold higher levels than in control samples (Fig. 8b, c). In leaf samples from plants maintained in the dark at 25 °C, the increase in HSP transcript abundance was much more marked so that after 12 h, the fold increases for the three test HSPs ranged from ca. 900- to 300-fold compared with controls maintained at 18 °C (Fig. 8a–c).

The expression patterns of these HSP genes were also determined by qRT-PCR in leaf samples maintained at 40 °C for 12 h and 24 h. Transcript abundance in leaf samples that had undergone acclimation (12 h light at 25 °C) was compared with leaf samples from non-acclimated plants (transferred directly from 18 to 40 °C; control samples C). It was clear that HSP expression at 40 °C was much higher in non-acclimated leaves than in fully acclimated leaves and that the expression levels for all three HSPs increased over the 24 h time course (Fig. 8d). Acclimated plants were also allowed to de-acclimate for 12 and 24 h at 18 °C prior to treatment at 40 °C. It was previously demonstrated that the acquired thermotolerance was lost during 12–24 h treatment at 18 °C (Fig. 2b). The expression profiles of the de-acclimated samples when exposed to 40 °C were broadly consistent with this finding, as the profile from leaves de-acclimated for 24 h was similar to the profile from non-acclimated plants (Fig. 8d). Plants de-acclimated for 12 h prior to 40 °C treatment exhibited an expression pattern intermediate between acclimated and non-acclimated plants (Fig. 8d).

## Discussion

### Acquired thermotolerance in potato

Despite the impact of moderately elevated temperatures on potato yield, detailed characterisation of the responses to such temperatures remain to be fully elucidated for this crop species. In this study, we focus on acquired thermotolerance in potato and demonstrate how acclimation at mildly elevated temperature has a strongly protective effect when plants are subsequently exposed to more severe episodes of temperature stress. Although acquired thermotolerance responses have been described for many model and crop species (Joshi et al. 1997; Senthil-Kumar et al. 2007; Larkindale and Vierling 2008; Finka et al. 2012; Song et al. 2012), we are unaware of any reports of this phenomenon in potato despite the long history of research on potato heat stress responses (Hetherington et al. 1983; Smillie et al. 1983). It is particularly striking that the acclimation temperature (25 °C) is lower than in most studies of this type, perhaps because potato is a cool climate crop. However, the range of temperatures used in this study is relevant to many areas where potato is cultivated.

### Gene and metabolite changes during acclimation and subsequent exposure to elevated temperature

Transfer of plants from 18 to 25 °C, during the acclimation process, resulted in significant restructuring of the potato leaf transcriptome. Within 2 h of exposure to 25 °C, there is a major increase in transcript abundance of heat shock factor-related transcripts and heat shock proteins (particularly, HSP17.6, HSC70 and HSP101). The transcript levels then generally remained at an elevated level throughout acclimation (Fig. 8a, Online Resource S3). In general, for temperate plants, elevated HSP transcript abundance occurs at temperatures in the range 32–35 °C (Vierling 1991) and so the large changes observed in potato at 25 °C emphasises the sensitivity of this species to elevated temperature. The major up-regulation of HSP expression in the dark at 25 °C compared with plants maintained at 18 °C (Fig. 8a–c) is an entirely novel finding of this study. It is interesting that the extremely high HSP expression level in dark treated plants was maintained on transfer to 40 °C, where the plants exhibited a complete lack of thermotolerance. Similarly, in plants transferred directly from 18 to 40 °C, HSP expression in leaves at the higher temperature was much higher than in plants that were acclimated. This observation implies that extremely high HSP expression levels are a reflection of severely stressed plants rather than an indication that the plants are protected from stress. We are unaware of previous examples of this pattern of HSP expression that suggests that where there is no acclimation, HSP expression is a response of “last resort”. The transcript profiles observed during acclimation also provide evidence of protein remodelling. There was a transient increase in several transcripts related to protein degradation (Fig. 6, Online Resource S3) that occurred as the levels of the free amino acids asparagine and glutamine increased (Online Resource S7). Transcript abundance of genes encoding protease inhibitors also increased transiently during acclimation, potentially contributing to protein remodelling by limiting the effects of proteases. The transcript patterns observed during acclimation strongly suggest there is a general growth arrest in leaves during this process. For example, there is a rapid decrease in transcripts related to cell wall modification including several encoding xyloglucan endotransglycosylases and cellulose synthase-like proteins. Similarly, the transcript levels of several auxin-responsive genes also decreased rapidly, a further indication of growth arrest (de Wit et al. 2016). Interestingly, the constitutive photomorphogenesis 1 (COP1) transcript levels decreased during the acclimation process. COP1 is involved in light signalling and is a negative regulator of the elongated hypocotyl 5 (HY5), a master regulator of plant growth (Ang et al. 1998). De-repression of HY5 results in an inhibition of growth, consistent with the general transcript profile.

Following transfer to 40 °C, both acclimated and non-acclimated plants exhibited similar changes in gene expression as illustrated by the observation that following analysis of variance based on time and prior acclimation treatment the overwhelming majority (> 9000) of transcripts were significantly altered in response to time at 40 °C irrespective of prior acclimation treatment. By contrast, only 39 transcripts exhibited variation in abundance based on acclimation, while 128 showed a time by acclimation interaction. Detailed analysis of these transcripts by hierarchical cluster analysis showed that in the acclimated plants, many of the changes in gene expression were delayed compared with the non-acclimated plants (Fig. 7, clusters C–F). These data highlight the need for an ordered and sequential response on transfer to high temperature, implying that certain events need to occur prior to others to allow the plant to cope with the high-temperature stress. This conclusion is supported by the observation that non-acclimated plants tended to show greater changes in the concentration of metabolites following transfer to higher temperatures, perhaps indicative of a greater dysregulation of metabolism (Online Resource S9). It was also interesting to note that non-acclimated plants exhibited an early stress transcriptome signature (Fig. 7, cluster A; Online Resource S11).

### Genetic variation in acquired thermotolerance responses

Within crop species, variation in acquired thermotolerance response between genotypes has previously been reported (reviewed in Senthil-Kumar et al. 2007). For example in wheat, expression of an HSP26 gene was associated with thermotolerance in recombinant inbred lines, but was not expressed in heat-susceptible recombinant lines (Joshi et al. 1997). In this study, we measured acquired thermotolerance in heat-tolerant and heat-susceptible genotypes from a biparental diploid potato population. Genotypes that produced poor tuber yield at elevated temperature generally (in 7 out of 8 genotypes tested) required acclimation at 25 °C to reduce membrane damage when exposed to elevated temperature. In contrast, heat-tolerant genotypes exhibited lower levels of membrane damage when exposed to 40 °C without acclimation treatment. As these genotypes were screened based on tuber yield, this indicates a link between the requirement for acclimation and storage organ yield, although genotype 242 did not follow this trend, indicating other mechanisms may also impact on heat tolerance. Similarly, there was considerable variation in the acclimation response of the wild potato species tested in this study. Whereas the accessions of VIO, BLV and IFO required acclimation at 25 °C to reduce membrane damage on exposure to 40 °C, the SPH, PNT, JAM, LPH and CHC did not require acclimation, indicating that there is considerable genetic variation for this trait. It will be of interest to correlate the thermotolerance response with the environment from which the wild species originate.

### Light requirement for acquired thermotolerance

The requirement for light during acclimation was clearly demonstrated in this study. We are unaware of any previous report of this requirement, although recent advances in understanding the integration of light and temperature signalling have been described (reviewed in Legris et al. 2017). It has also been demonstrated that light is required for cold acclimation responses in Arabidopsis (Catalá et al. 2011). Integration of low temperature and light signalling involves the COP1/HY5 pathway and so there may be parallels with high-temperature acclimation. It is now clear that the phytochrome B (PHYB) photoreceptor also functions as a temperature sensor integrating light and temperature signals (Legris et al. 2016; Jung et al. 2016). PHYB exists as dimers in inactive Pr and active Pfr forms. Red light drives the conversion of Pr to Pfr, whereas far-red light converts it back. Pfr can also be converted to Pr in the dark by a light independent thermal reversion process. The dark-reversion light-independent reaction was also shown to be accelerated by elevated temperature. Thus, in the dark, the amount of active Pfr is temperature dependent. Downstream of PHYB, two interconnected signalling networks have been shown to operate in the control of plant growth: the PIF4 branch and another branch involving COP1 and HY5 (reviewed in Legris et al. 2017). These signalling networks exert major control over many processes in plant development.

Although it may be possible that pathways downstream of PHYB may provide the regulatory mechanisms for acquired thermotolerance, another possibility is that retrograde signals derived from the chloroplast are involved either independently or in concert with the PHYB-mediated response. The importance of retrograde signalling in control of nuclear gene expression is an emerging theme in plant research with the recent demonstration that chloroplasts can fine-tune physiological responses to stresses including drought (Pornsiriwong et al. 2017). Our results indicate a slight increase in the oxidation status of both the ascorbate and glutathione pools during the acclimatory process (Fig. 4), indicative of an oxidative signal that could be involved in the priming response (Foyer et al. 2017). The observation that maximal quantum efficiency of PSII was maintained during acclimation in the light (Fig. 3b) indicates that the leaf did not experience severe oxidative stress during the acclimatory period. Surprisingly, acclimation in the dark at 25 °C resulted in a significant loss of the Fv/Fm ratio suggesting that in the absence of light, the mildly elevated temperature resulted in significant damage to the stability and operation of PSII. Therefore, it is also possible that transfer directly to 40 °C or acclimation in the dark at 25 °C prior to transfer results in extensive PSII damage, resulting in the production of an oxidative stress signal leading to cell death in non-acclimated plants. However, it should also be noted that plants acclimated in the light for 12 h then experienced 40 °C for the first 12 h also in the light, i.e. they experience a 24 h light cycle. This may induce a stress response, which could lead to cross tolerance. Although we were unable to detect a light stress signature in the present work, this possibility requires further analysis.

Further work on acquired thermotolerance in potato will be required to gain a detailed mechanistic understanding of the response. The availability of transgenic potato lines in which the expression level of *PHYB* is manipulated will provide a useful experimental platform for dissecting how the process is regulated. Comparison of germplasm identified in this study that exhibits different requirements for acquired thermotolerance may enable a greater understanding of the trait and ultimately identify useful markers that could be deployed as part of a conventional breeding programme.

#### *Author contribution statement*

AT-M, LJMD, CEB, WM, CW, JAM and CP performed the experiments; PEH supervised the microarray experiments, AT-M, LJMD, CEB, WM, PEH, RDH and MT interpreted the results and wrote the research paper; RDH and MT designed and supervised the research work.

## Electronic supplementary material

Below is the link to the electronic supplementary material.

Supplementary material 1 (DOCX 86 kb)

Supplementary material 2 (DOCX 17 kb)

Supplementary material 3 (XLSX 90 kb)

Supplementary material 4 (DOCX 142 kb)

Supplementary material 5 (XLSX 24 kb)

Supplementary material 6 (XLSX 26 kb)

Supplementary material 7 (DOCX 212 kb)

Supplementary material 8 (XLSX 38 kb)

Supplementary material 9 (PDF 1166 kb)

Supplementary material 10 (DOCX 575 kb)

Supplementary material 11 (XLSX 37 kb)

Supplementary material 12 (DOCX 88 kb)

## Abbreviations

HSP: Heat shock protein
ROS: Reactive oxygen species
