# Correction to: A reversible light- and genotype-dependent acquired thermotolerance response protects the potato plant from damage due to excessive temperature

**DOI:** 10.1007/s00425-018-2900-3

**Published:** 2018-05-03

**Authors:** Almudena Trapero-Mozos, Laurence J. M. Ducreux, Craita E. Bita, Wayne Morris, Cosima Wiese, Jenny A. Morris, Christy Paterson, Peter E. Hedley, Robert D. Hancock, Mark Taylor

**Affiliations:** 1 School of Biology, Biomolecular Sciences Building, University of St Andrews, North Haugh, St Andrews, Fife, Y16 9ST UK; 2 Cell and Molecular Sciences, The James Hutton Institute, Invergowrie, Dundee, DD2 5DA UK; 3 College of Arts and Sciences, Misericordia University, 301 Lake Street, Dallas, PA 18612 USA

## Correction to: Planta 10.1007/s00425-018-2874-1

The article A reversible light- and genotype-dependent acquired thermotolerance response protects the potato plant from damage due to excessive temperature, written by Almudena Trapero-Mozos, Laurence J. M. Ducreux, Craita E. Bita, Wayne Morris, Cosima Wiese, Jenny A. Morris, Christy Paterson, Peter E. Hedley, Robert D. Hancock, and Mark Taylor, was originally published electronically on the publisher’s internet portal (currently SpringerLink) on 8 March 2018 without open access. With the author(s)’ decision to opt for Open Choice the copyright of the article changed on 30 April 2018 to © The Author(s) 2018 and the article is forthwith distributed under the terms of the Creative Commons Attribution 4.0 International License (http://creativecommons.org/licenses/by/4.0/), which permits use, duplication, adaptation, distribution and reproduction in any medium or format, as long as you give appropriate credit to the original author(s) and the source, provide a link to the Creative Commons license and indicate if changes were made.

The ​original ​article ​has ​been ​corrected.

