# Supplementary material for: A reversible light- and genotype-dependent acquired thermotolerance response protects the potato plant from damage due to excessive temperature
Source: Planta. 2018 Mar 8;247(6):1377–92. doi: 10.1007/s00425-018-2874-1 (PMC5945765; doi:10.1007/s00425-018-2874-1)
Supplement: Supplementary file 1 — Supplementary material 1 (DOCX 86 kb) [file 425_2018_2874_MOESM1_ESM.docx]

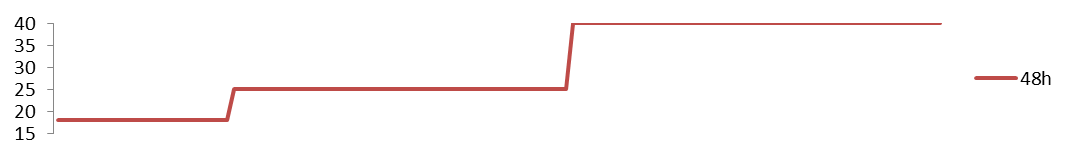

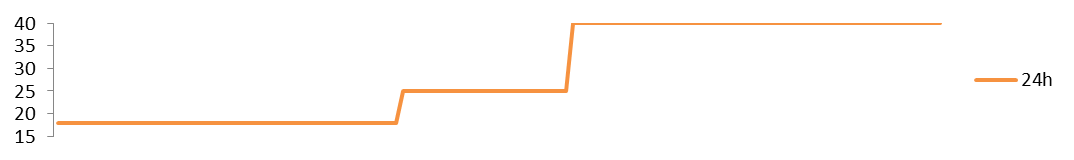

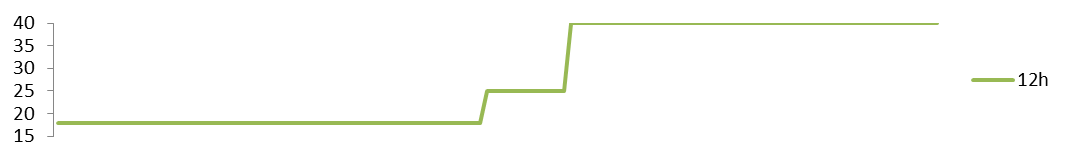

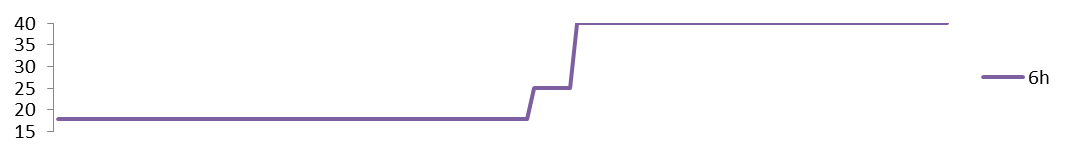

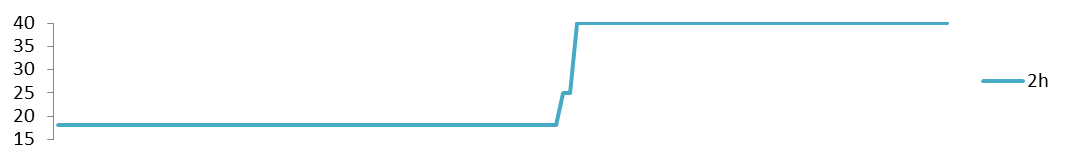

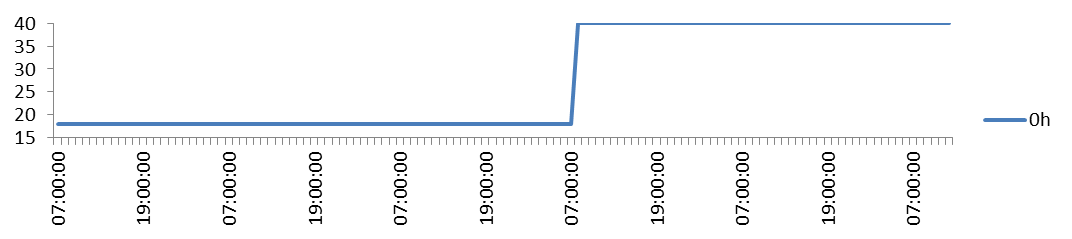


Temperature °C

*****

*****

*****

*****

*****

*****

*****

*****

*****

Watering

Lights off

Heat Stress 40 ^0^C

*****

**Online Resource S1** **a** Thermotolerance assay – timecourse experiments. Prior to acclimation treatment, plants were moved to a growth chamber maintained at 18°C, 12h day length, for one week. To induce thermotolerance, plants were then transferred to 25 °C (acclimation treatment) under constant light for different periods of time (0, 2, 6, 12, 24 or 48 h), prior to heat stress at 40 °C.


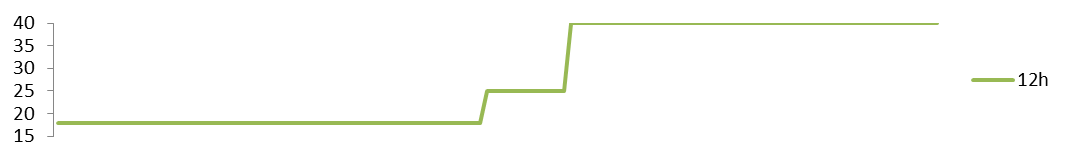


Temperature °C


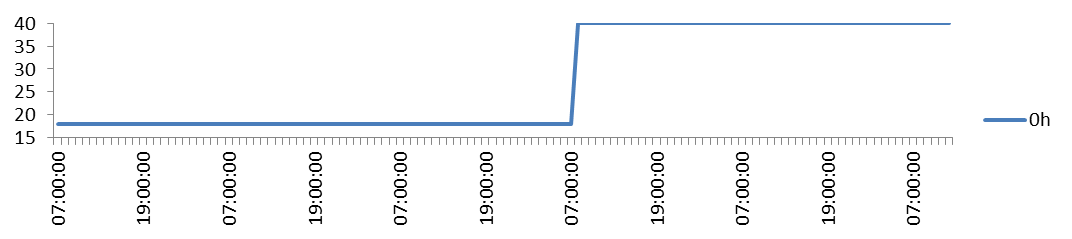


**Onlilne Resource S1 b** Standard thermotolerance assay. Prior to acclimation treatment, plants were moved to a growth chamber maintained at 18°C, 12h day length, for one week. To induce thermotolerance, plants were then transferred to 25 °C (acclimation treatment – control plants were left at 18°C) under constant light, prior to heat stress at 40 °C.

**Planta**

**A reversible light and genotype dependent acquired thermotolerance response protects the potato plant from excessive temperature**

Almudena Trapero-Mozos^1*^, Laurence JM Ducreux^2*^, Craita E Bita^2*^, Wayne Morris^2^, Cosima Wiese^3^, Jenny A Morris^2^, Christy Paterson^2^, Peter E Hedley^2^, Robert D Hancock^2*^, Mark Taylor^2*^

Corresponding author: [mark.taylor@hutton.ac.uk](mailto:mark.taylor@hutton.ac.uk)

Cell & Molecular Sciences, The James Hutton Institute, Invergowrie, Dundee DD2 5DA, United Kingdom.

*****

Watering

Lights off

*****

*****

*****

*****

*****

*****

*****

*****

Heat Stress 40 ^0^C

*****
