# Supplementary material for: A reversible light- and genotype-dependent acquired thermotolerance response protects the potato plant from damage due to excessive temperature
Source: Planta. 2018 Mar 8;247(6):1377–92. doi: 10.1007/s00425-018-2874-1 (PMC5945765; doi:10.1007/s00425-018-2874-1)
Supplement: Supplementary file 2 — Supplementary material 2 (DOCX 17 kb) [file 425_2018_2874_MOESM2_ESM.docx]

**Online Resource S2** Tuber yield of 06H1 genotypes at low temperature and high temperature

| **Clone Identifier** | **Classified** | **Total Fresh Weight (g) 28^0^C** | **Total Fresh Weight (g) 22^0^C** |
| --- | --- | --- | --- |
| **50** | HEAT TOLERANT | 8.0 | 1.9 |
| **153** | HEAT TOLERANT | 4.5 | 5.6 |
| **202** | HEAT SENSITIVE | 0.0 | 3.0 |
| **206** | HEAT SENSITIVE | 0.0 | 3.1 |
| **242** | HEAT SENSITIVE | 0.0 | 2.8 |
| **278** | HEAT SENSITIVE | 0.0 | 4.7 |
| **289** | HEAT TOLERANT | 4.6 | 6.2 |
| **295** | HEAT TOLERANT | 3.4 | 3.0 |

**Planta**

**A reversible light and genotype dependent acquired thermotolerance response protects the potato plant from excessive temperature**

Almudena Trapero-Mozos^1*^, Laurence JM Ducreux^2*^, Craita E Bita^2*^, Wayne Morris^2^, Cosima Wiese^3^, Jenny A Morris^2^, Christy Paterson^2^, Peter E Hedley^2^, Robert D Hancock^2*^, Mark Taylor^2*^

Corresponding author: [mark.taylor@hutton.ac.uk](mailto:mark.taylor@hutton.ac.uk)

Cell & Molecular Sciences, The James Hutton Institute, Invergowrie, Dundee DD2 5DA, United Kingdom.
