# Supplementary material for: A reversible light- and genotype-dependent acquired thermotolerance response protects the potato plant from damage due to excessive temperature
Source: Planta. 2018 Mar 8;247(6):1377–92. doi: 10.1007/s00425-018-2874-1 (PMC5945765; doi:10.1007/s00425-018-2874-1)
Supplement: Supplementary file 4 — Supplementary material 4 (DOCX 142 kb) [file 425_2018_2874_MOESM4_ESM.docx]

**Online Resource S4** Venn diagram of significantly differentially abundant leaf transcripts following 2, 6 and 12 h acclimation at 25 ^0^C**.** Transcripts significantly differentially abundant at 2, 6 and 12 h were estimated by pairwise comparison with transcript abundance immediately following transfer to 25°C. Venn diagram indicates the number of transcripts expressed at one or more time points and the size of circles is adjusted for the total number of transcripts.

**Planta**

**A reversible light and genotype dependent acquired thermotolerance response protects the potato plant from excessive temperature.**

Almudena Trapero-Mozos1*, Laurence JM Ducreux2*, Craita E Bita2*, Wayne Morris2, Cosima Wiese3, Jenny A Morris2, Christy Paterson2, Peter E Hedley2, Robert D Hancock2*, Mark Taylor2*

Corresponding author: mark.taylor@hutton.ac.uk

Cell & Molecular Sciences, The James Hutton Institute, Invergowrie, Dundee DD2 5DA, United Kingdom.
