# Supplementary material for: A reversible light- and genotype-dependent acquired thermotolerance response protects the potato plant from damage due to excessive temperature
Source: Planta. 2018 Mar 8;247(6):1377–92. doi: 10.1007/s00425-018-2874-1 (PMC5945765; doi:10.1007/s00425-018-2874-1)
Supplement: Supplementary file 7 — Supplementary material 7 (DOCX 212 kb) [file 425_2018_2874_MOESM7_ESM.docx]

**Online Resource 7** Relative concentration of leaf metabolites significantly changing in abundance during acclimation. Leaves were harvested and relative metabolite concentration estimated by GC/MS as described. Data are presented as mean metabolite concentration relative to the respective polar or non-polar internal standard ± SE, *n* = 3. Different letters indicate significantly different values as determined by one-way ANOVA with Tukey’s protected LSD test (*P*<0.1). Sucrose (**a**), sorbitol (**b**), mannitol (**c**), galactinol (**d**), asparagine (**e**), glutamine (**f**), allantoin (**g**), tetracosanol (**h**), nonacosanol (**i**)

**Planta**

**A reversible light and genotype dependent acquired thermotolerance response protects the potato plant from excessive temperature.**

Almudena Trapero-Mozos1*, Laurence JM Ducreux2*, Craita E Bita2*, Wayne Morris2, Cosima Wiese3, Jenny A Morris2, Christy Paterson2, Peter E Hedley2, Robert D Hancock2*, Mark Taylor2*

Corresponding author: mark.taylor@hutton.ac.uk

Cell & Molecular Sciences, The James Hutton Institute, Invergowrie, Dundee DD2 5DA, United Kingdom.
