# Supplementary material for: A reversible light- and genotype-dependent acquired thermotolerance response protects the potato plant from damage due to excessive temperature
Source: Planta. 2018 Mar 8;247(6):1377–92. doi: 10.1007/s00425-018-2874-1 (PMC5945765; doi:10.1007/s00425-018-2874-1)
Supplement: Supplementary file 9 — Supplementary material 9 (PDF 1166 kb) [file 425_2018_2874_MOESM9_ESM.pdf]

1. Metabolites significantly influenced by time

Sugars and related carbohydrates

Glucose peak 1

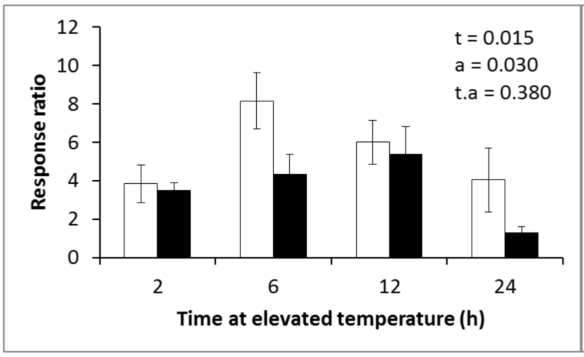

Glucose peak 2

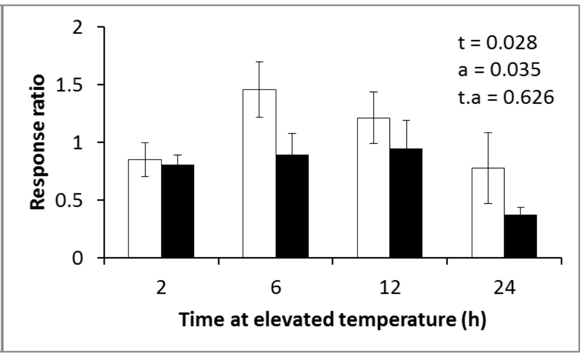

Unoximated glucose peak 1

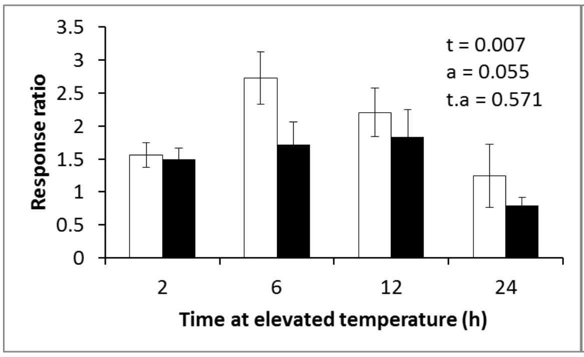

Unoximated glucose peak 2

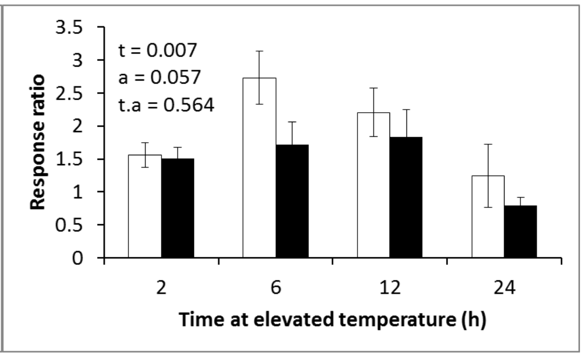

Fructose peak 1

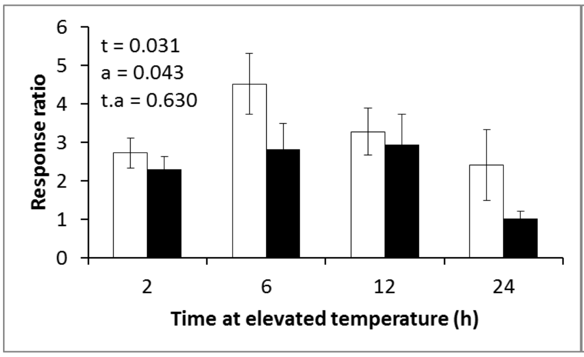

Fructose peak 2

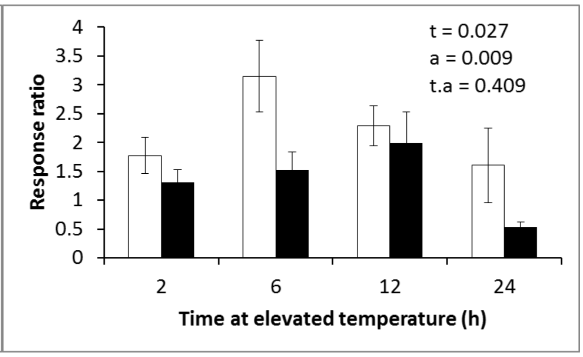

Glactose

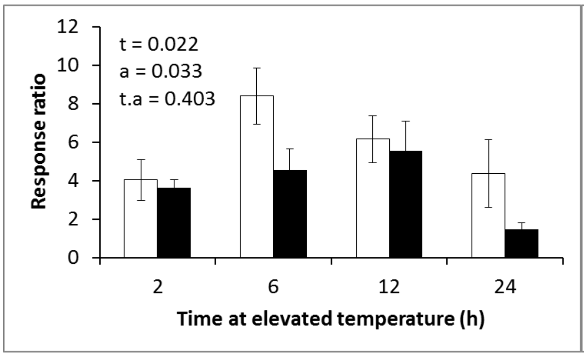

Sucrose

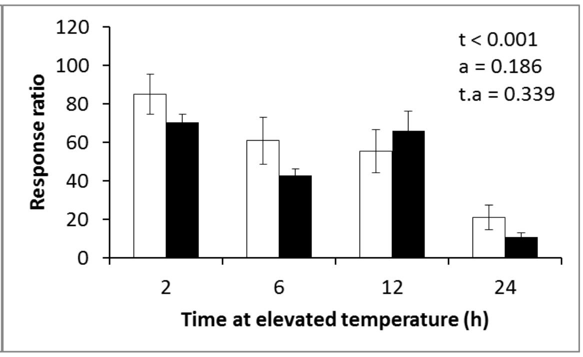

Maltose

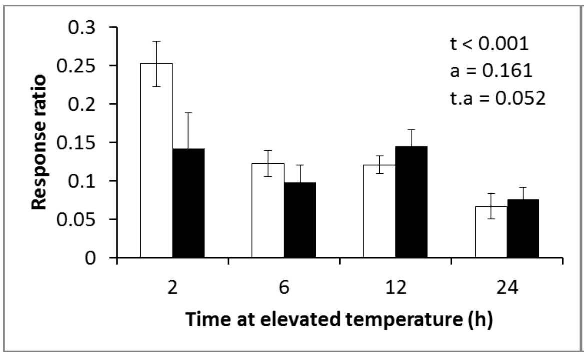

Mannitol

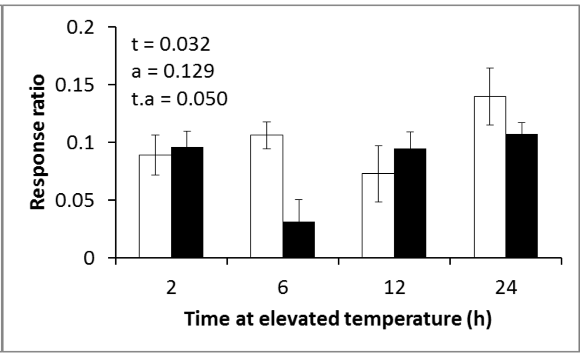

Sorbitol

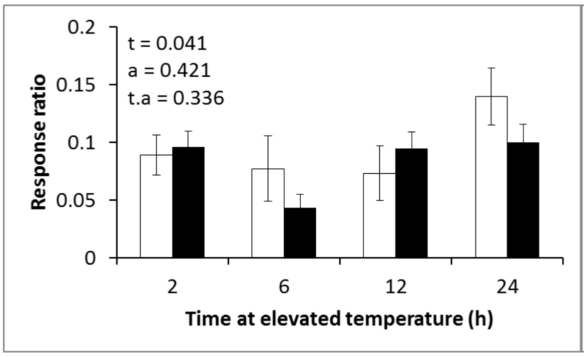

Dihydroxydihydrofuranone

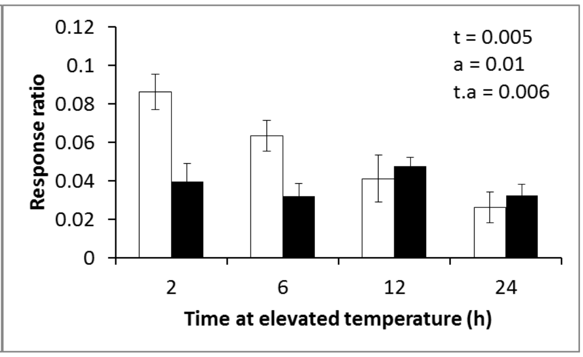

Organic acids

Citrate

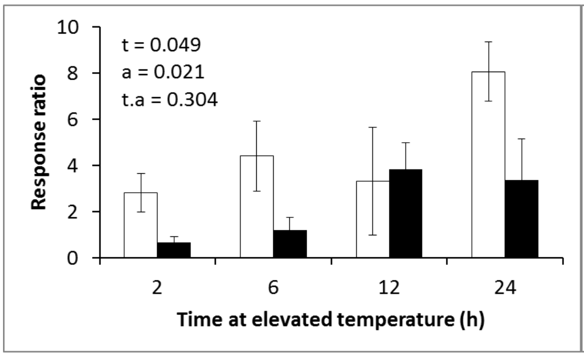

Malate

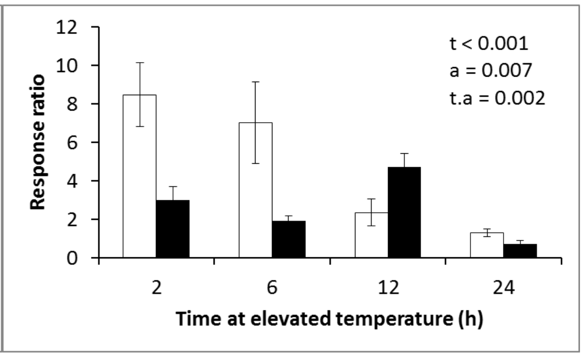

Threonate

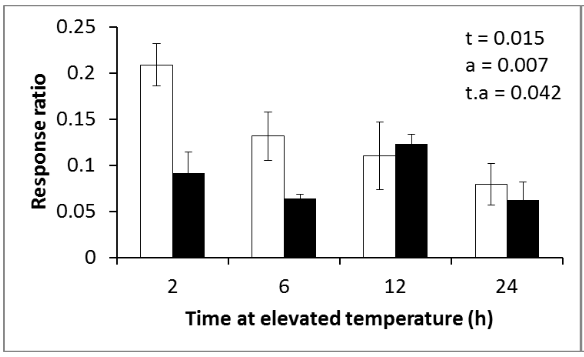

Trihydroxypentanoate

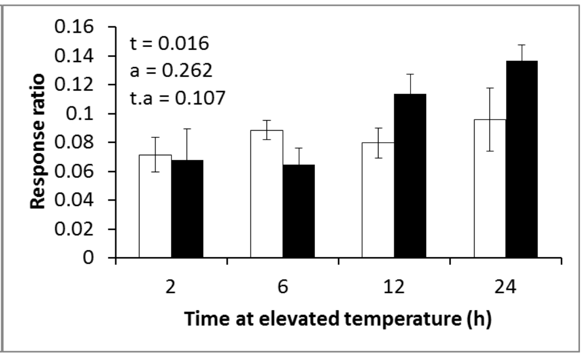

Quinate

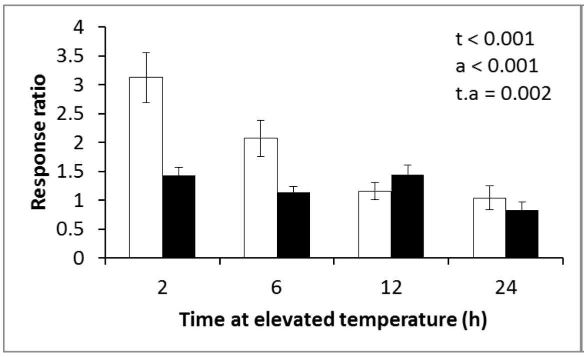

Glycerate

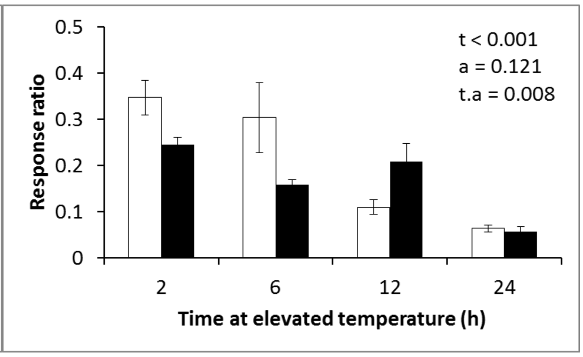

Amino acids and amines

Asparagine peak 1

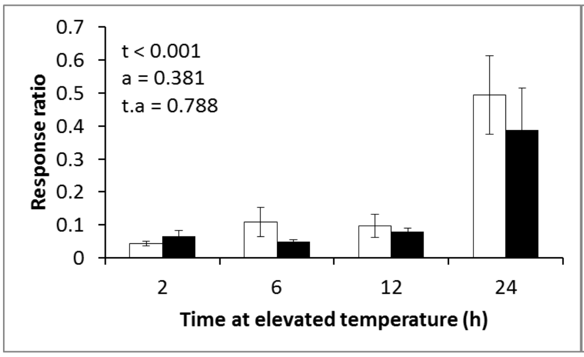

Asparagine peak 2

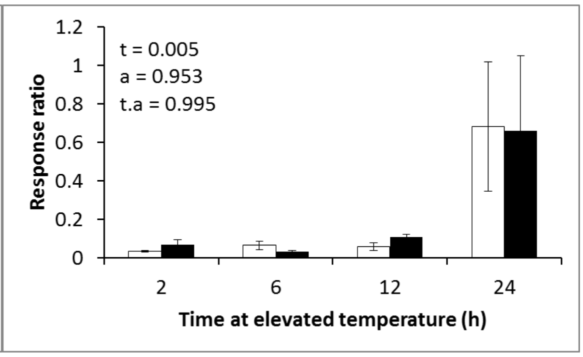

Glutamine peak 1

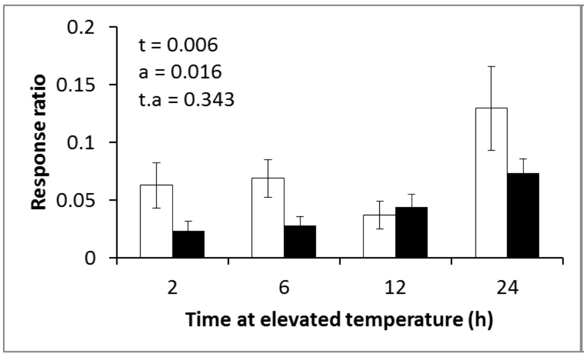

Glutamine peak 2

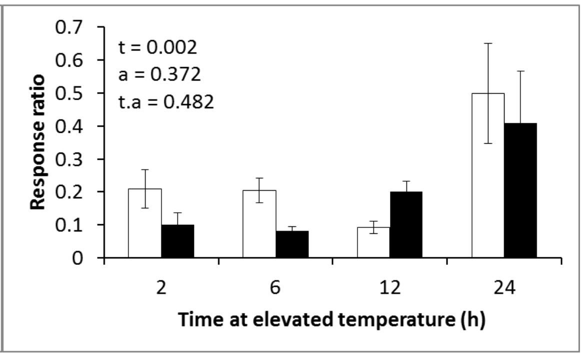

Serine

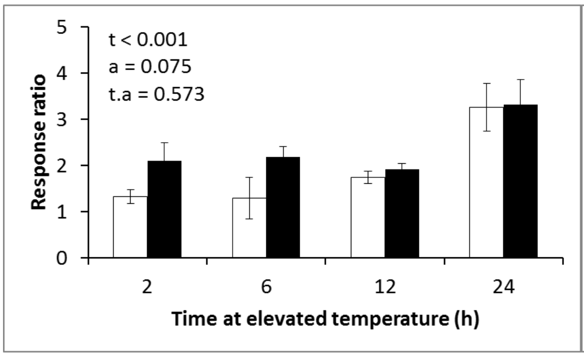

Glycine

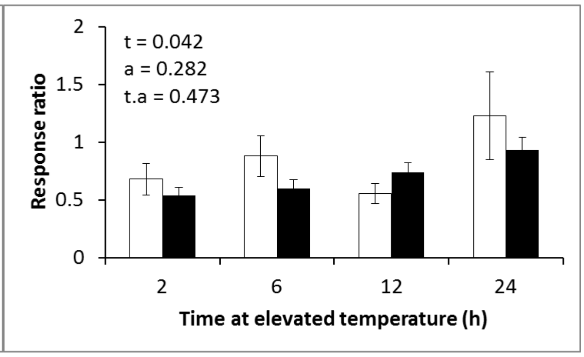

Phenylalanine

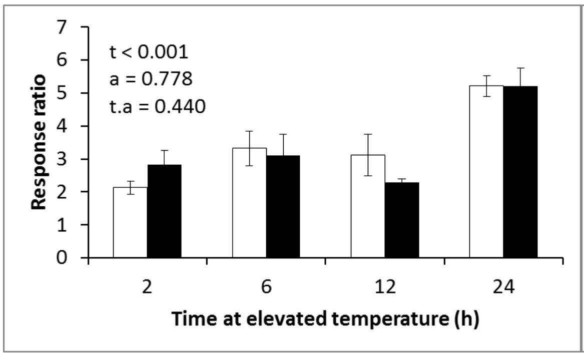

Tyrosine

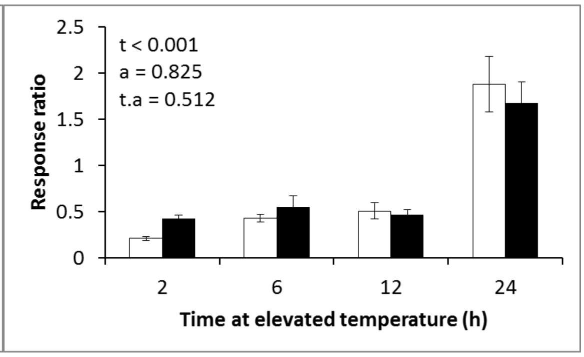

Valine

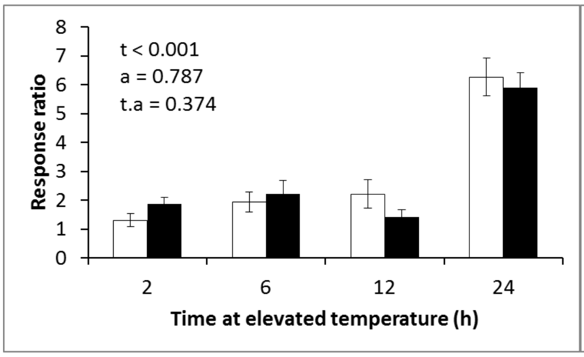

Leucine

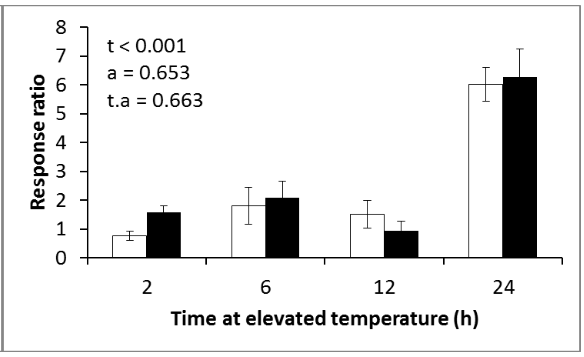

Isoleucine

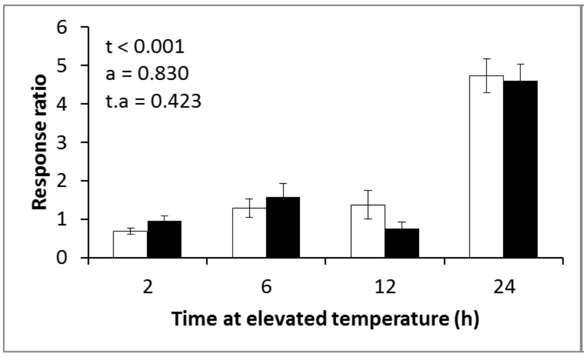

Lysine

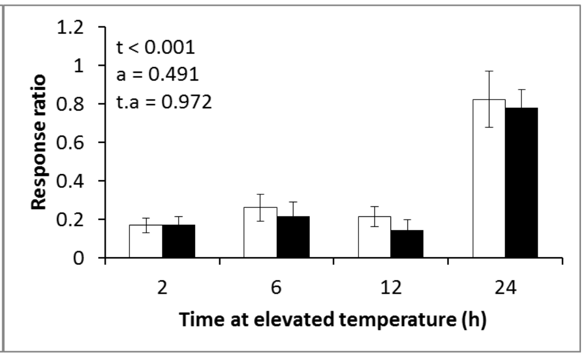

Threonine

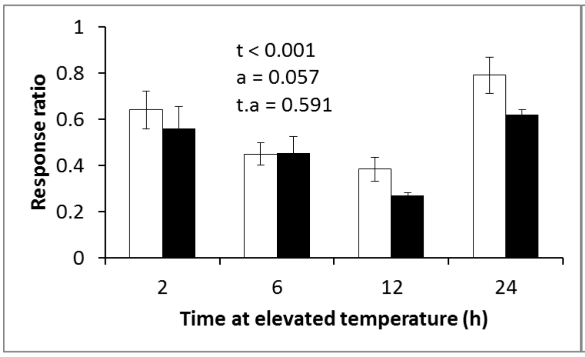

Aspartate

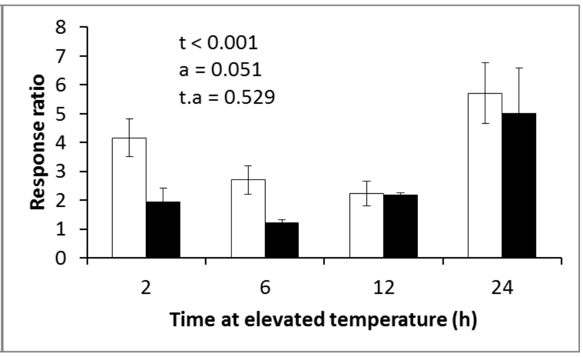

Spermidine

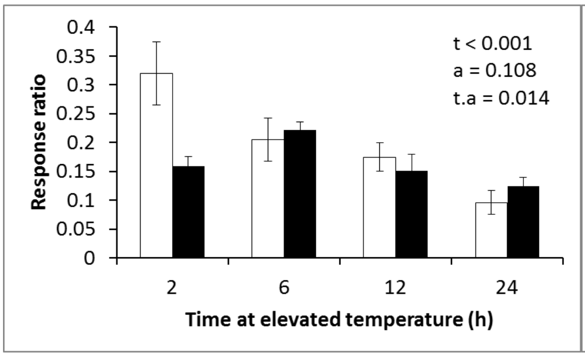

Putrescine

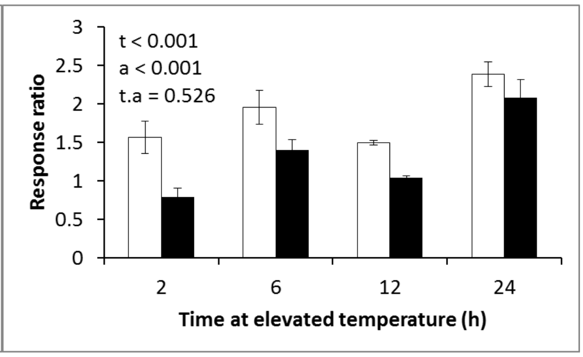

Allantoin peak 1

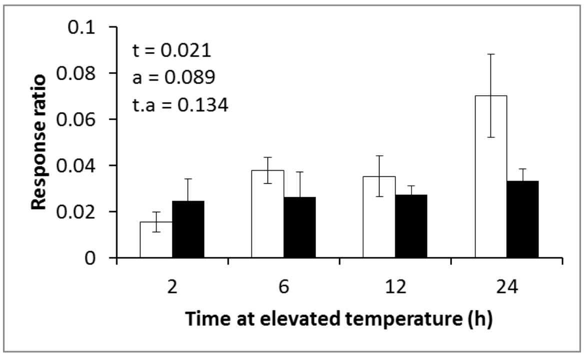

Fatty Acids

Tetradecanoic acid

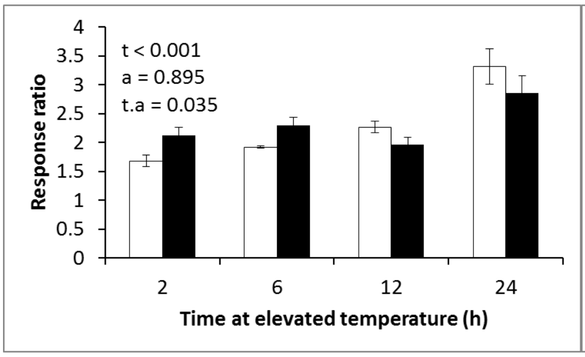

Eicosanoic acid

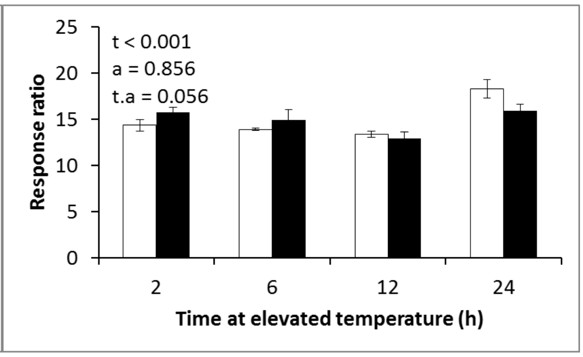

Docosanoic acid

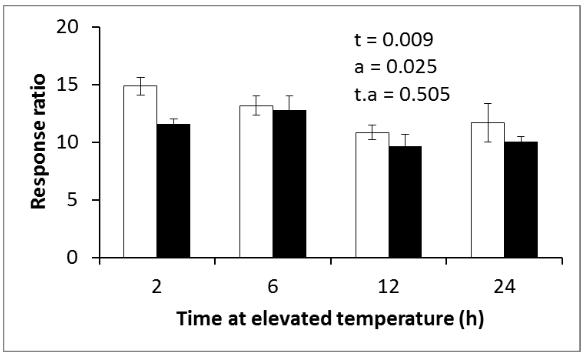

Fatty alcohols

Tetracosanol

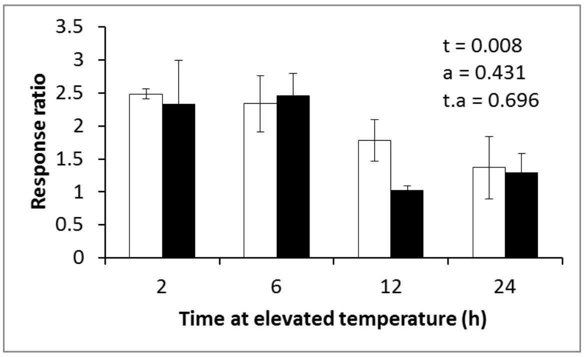

Phytosterols

Stigmastadienol

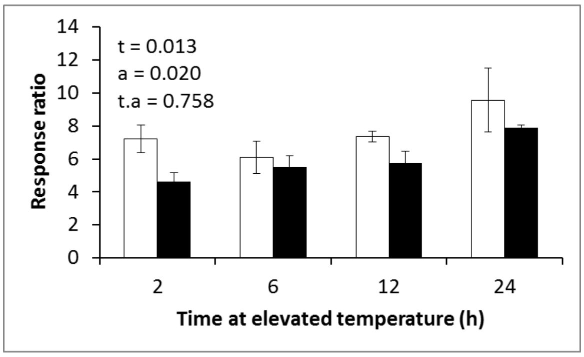

2. Metabolites significantly influenced by acclimation treatment

Sugars and related carbohydrates

Glucose peak 1

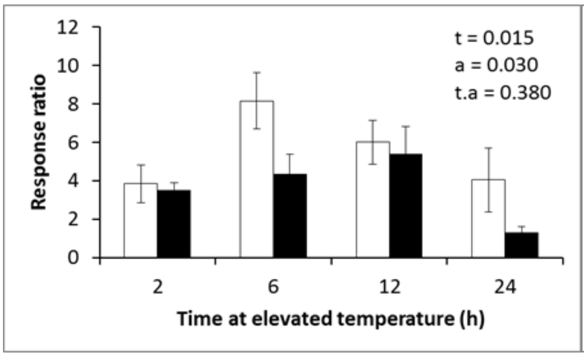

Glucose peak 2

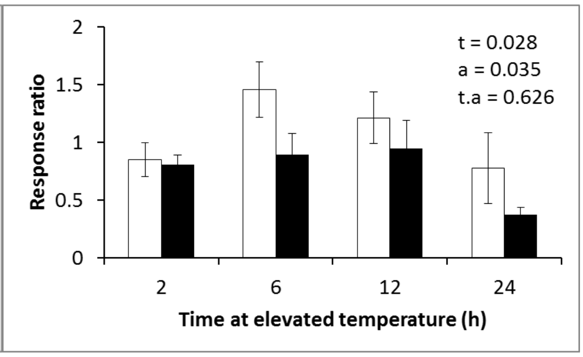

Fructose peak 1

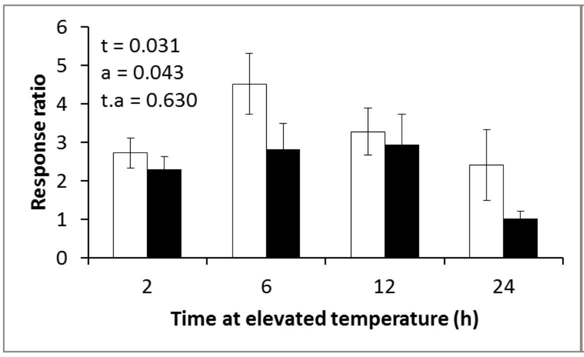

Fructose peak 2

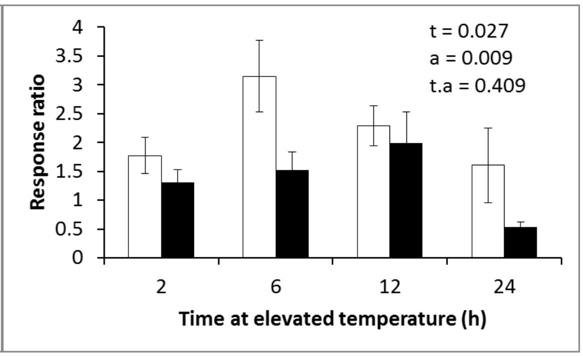

Galactose

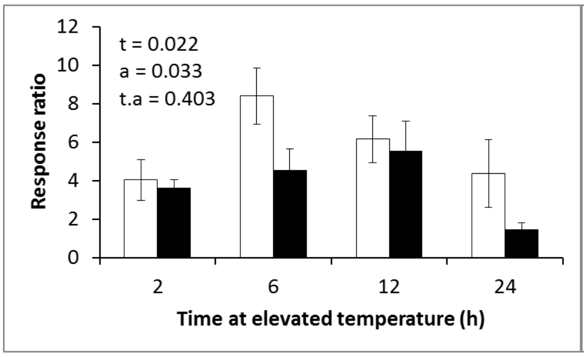

Dihydroxydihydrofuranone

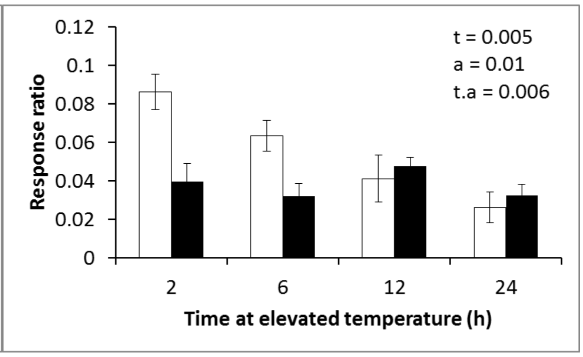

Organic acids

Succinate

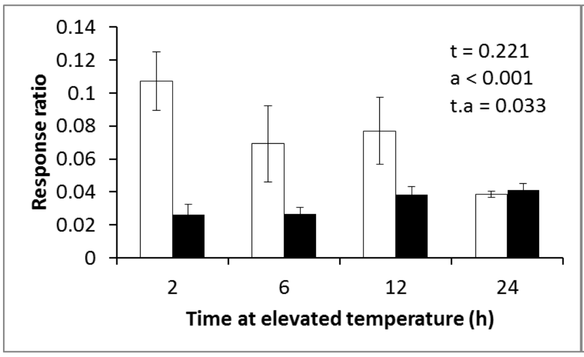

Citrate

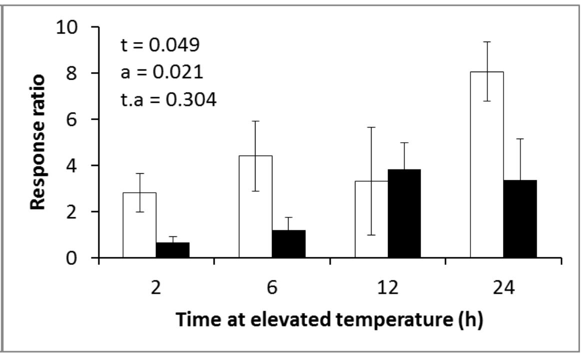

Malate

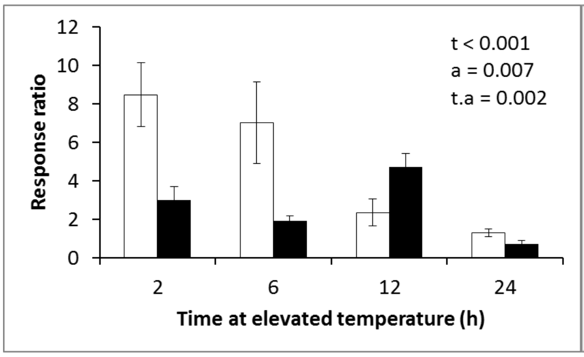

Threonate

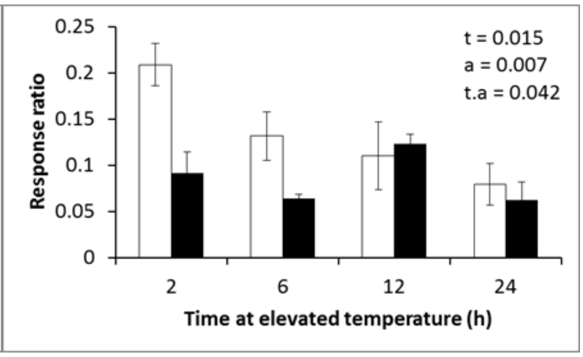

Quinate

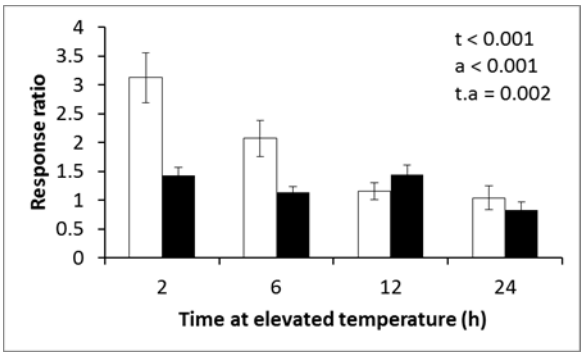

Amino acids and amines

Glutamine peak 1

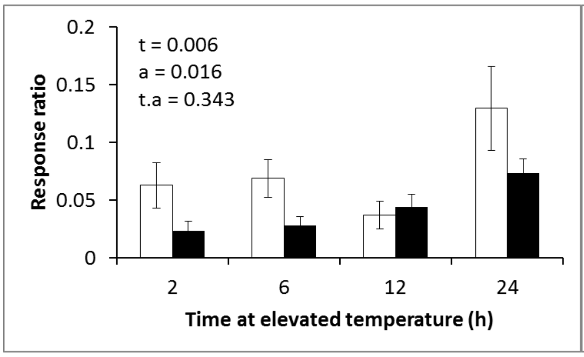

Putrescine

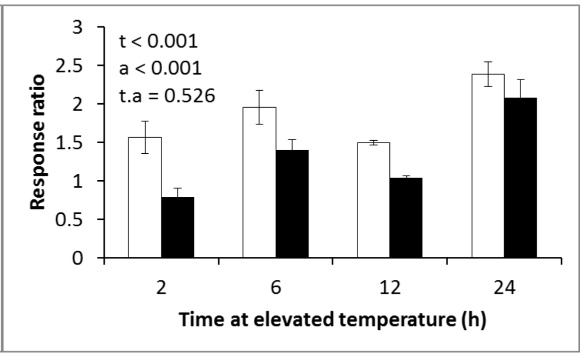

Phenolic acids

Chlorogenic acid

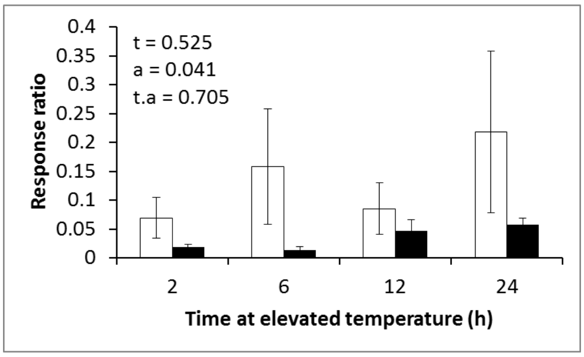

Fatty acids

Octadecenoic acid

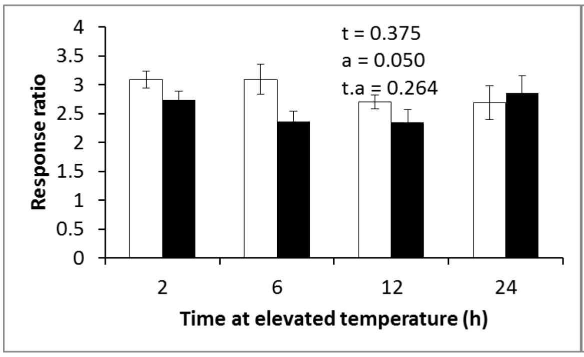

Linoleic acid

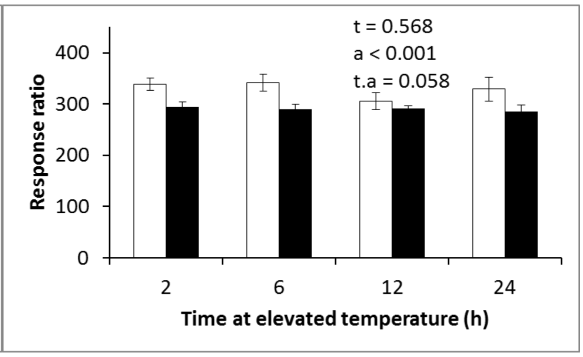

Docosanoic acid

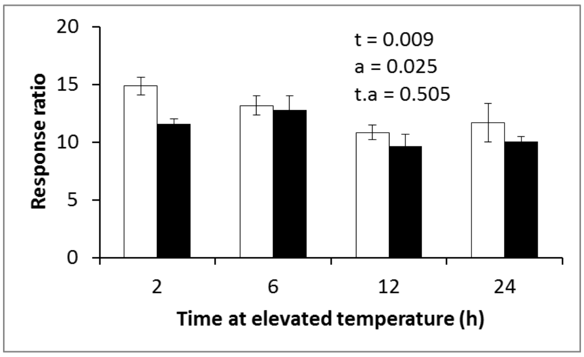

Phytosterols

Stigmastadienol

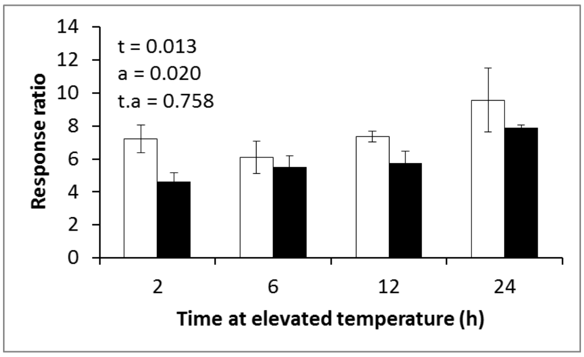

3. Metabolites significantly influenced by an interaction between time and acclimation

Sugars and related carbohydrates

Mannitol

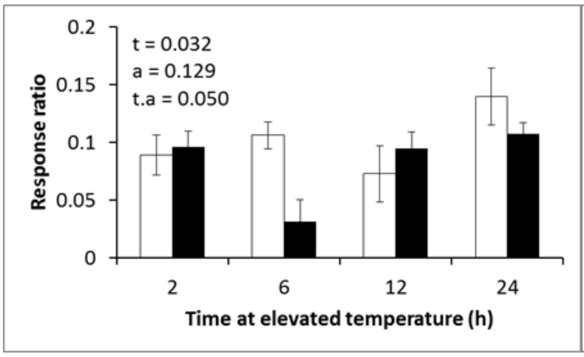

Dihydroxydihydrofuranone

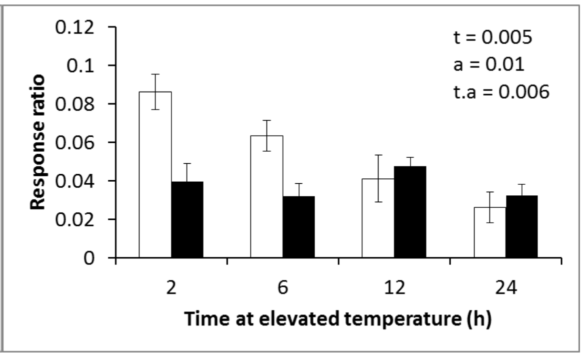

Organic acids

Succinate

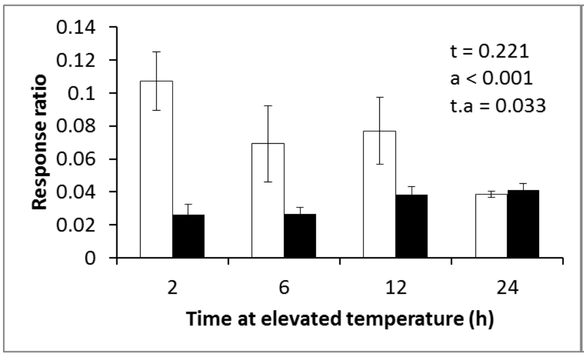

Malate

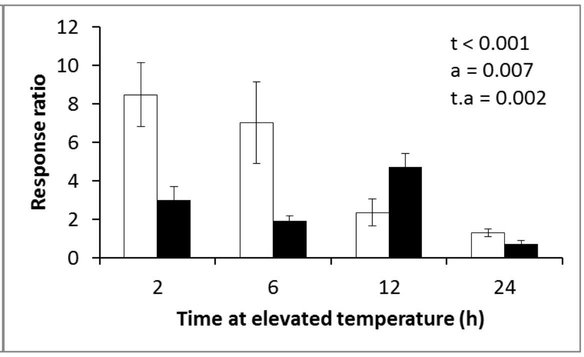

Threonate

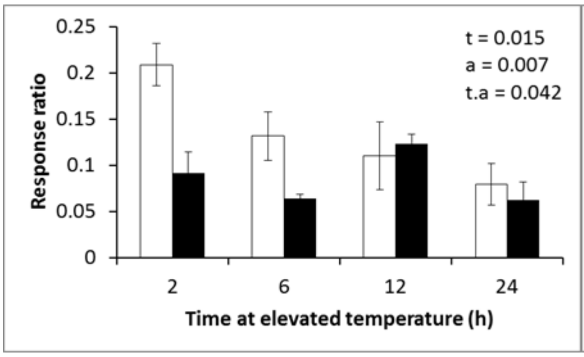

Quinate

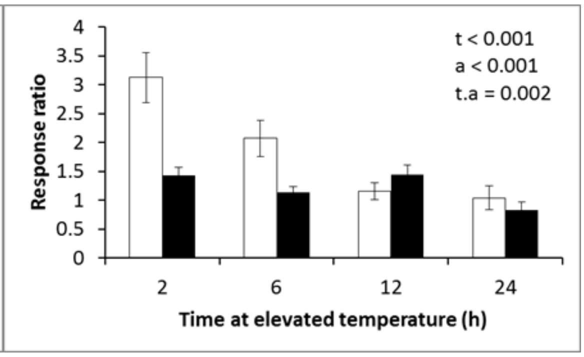

Glycerate

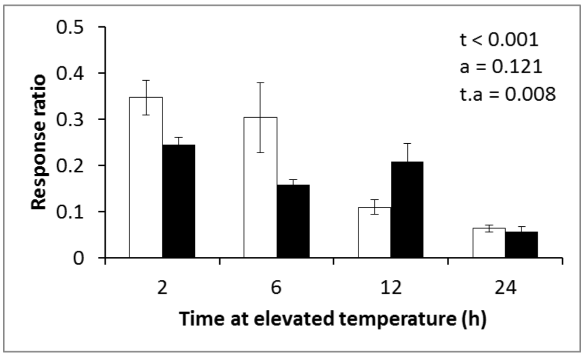

Amino acids and amines

Spermidine

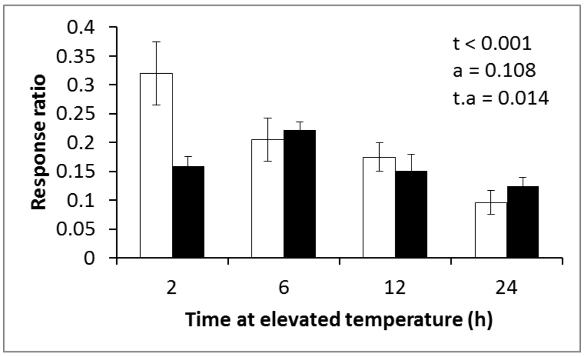

Fatty acids

Tetradecanoic acid

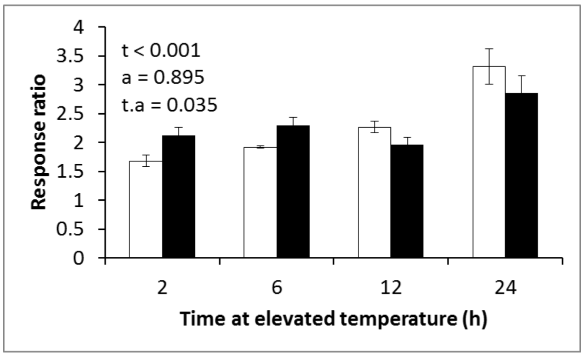

Fatty alcohols

Heneicosanol

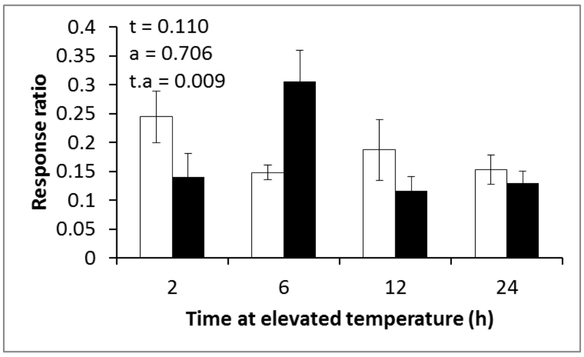

Phytosterols

Avanasterol

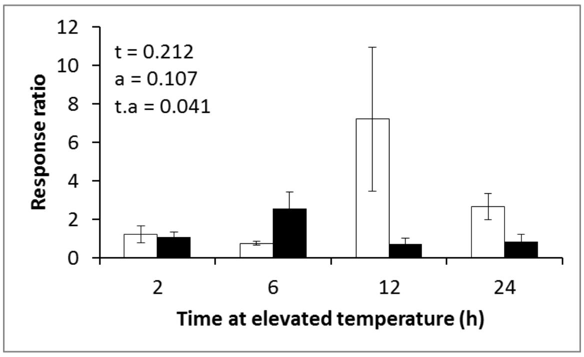

Online Resource 8 Relative concentration of metabolites in acclimated and non-acclimated potato leaves following transfer to 40 °C

Concentrations of metabolites significantly altered by acclimation treatment, time at high temperature or an interaction of the two factors are indicated based in GC/MS peak area relative to the peak area of appropriate polar or non-polar internal standards (response ratio). Data for non-acclimated (open bars) or acclimated (closed bars) are indicated as mean ± SE (n = 3). P-values for two-way ANOVA based on time at 40 °C (t), prior acclimation treatment (a) or an interaction of the two factors (t.a) are indicated.

A Reversible Light and Genotype Dependent Acquired Thermotolerance Response Protects the Potato Plant from Excessive Temperature.

Planta

Almudena Trapero-Mozos[1*], Laurence JM Ducreux[2*], Craita E Bita[2*], Wayne Morris[2], Cosima Wiese[3], Jenny A Morris[2], Christy Paterson[2], Peter E Hedley[2], Robert D Hancock[2*], Mark Taylor[2*]

Corresponding author: mark.taylor@hutton.ac.uk

Cell & Molecular Sciences, The James Hutton Institute, Invergowrie, Dundee DD2 5DA, United Kingdom.
