# Supplementary material for: A reversible light- and genotype-dependent acquired thermotolerance response protects the potato plant from damage due to excessive temperature
Source: Planta. 2018 Mar 8;247(6):1377–92. doi: 10.1007/s00425-018-2874-1 (PMC5945765; doi:10.1007/s00425-018-2874-1)
Supplement: Supplementary file 10 — Supplementary material 10 (DOCX 575 kb) [file 425_2018_2874_MOESM10_ESM.docx]

**Online Resource S10** PageMan representation of gene expression in acclimated (ACC) and unacclimated (NAC) potato leaves following different periods of exposure to 40°C during a 24 h time course. Values presented are log2 fold changes for the differentially expressed genes with respect to time (9330 probes) subjected to a Wilcoxon rank sum test to determine if specific ontologies were up-regulated (blue) or down-regulated (red) relative to the median fold change of all genes.

**Planta**

**A reversible light and genotype dependent acquired thermotolerance response protects the potato plant from excessive temperature.**

Almudena Trapero-Mozos1*, Laurence JM Ducreux2*, Craita E Bita2*, Wayne Morris2, Cosima Wiese3, Jenny A Morris2, Christy Paterson2, Peter E Hedley2, Robert D Hancock2*, Mark Taylor2*

Corresponding author: mark.taylor@hutton.ac.uk

Cell & Molecular Sciences, The James Hutton Institute, Invergowrie, Dundee DD2 5DA, United Kingdom.
