# Supplementary material for: A reversible light- and genotype-dependent acquired thermotolerance response protects the potato plant from damage due to excessive temperature
Source: Planta. 2018 Mar 8;247(6):1377–92. doi: 10.1007/s00425-018-2874-1 (PMC5945765; doi:10.1007/s00425-018-2874-1)
Supplement: Supplementary file 12 — Supplementary material 12 (DOCX 88 kb) [file 425_2018_2874_MOESM12_ESM.docx]

**Online Resource 12.** Acclimation treatment microarray data validation. Upper panel: HSP17.6, HSC70 and HSP101 transcript level changes in the microarray experiment during different plant acclimation treatments at 25 ^0^C (2hA, 6hA, 12hA) in light conditions. Lower panel: relative gene expression changes in HSP17.6, HSC70 and HSP101 transcript levels measured by RT-PCR during different plant acclimation treatments at 250C (2hA, 6hA, 12hA) under light conditions. Gene expression is calculated relative to standard conditions (plants maintained at 18 ^0^C). Data is presented as the mean and standard error of the mean for independent experiments (*n* = 3)

**A Reversible Light and Genotype Dependent Acquired Thermotolerance Response Protects the Potato Plant from Excessive Temperature.**

**Planta**

Almudena Trapero-Mozos1*, Laurence JM Ducreux2*, Craita E Bita2*, Wayne Morris2, Cosima Wiese3, Jenny A Morris2, Christy Paterson2, Peter E Hedley2, Robert D Hancock2*, Mark Taylor2*

Corresponding author: mark.taylor@hutton.ac.uk

Cell & Molecular Sciences, The James Hutton Institute, Invergowrie, Dundee DD2 5DA, United Kingdom.
